# Prediction of Protein Structure Using Surface Accessibility Data

**DOI:** 10.1002/anie.201604788

**Published:** 2016-08-25

**Authors:** Christoph Hartlmüller, Christoph Göbl, Tobias Madl

**Affiliations:** ^1^ Center for Integrated Protein Science MunichTechnische Universität MünchenDepartment of ChemistryLichtenbergstrasse 485748GarchingGermany; ^2^ Institute of Structural BiologyHelmholtz Zentrum MünchenIngolstädter Landstrasse 185764NeuherbergGermany; ^3^ Institute of Molecular Biology & BiochemistryCenter of Molecular MedicineMedical University of Graz8010GrazAustria

**Keywords:** CS-Rosetta, NMR spectroscopy, paramagnetic relaxation, protein structure prediction, structural biology

## Abstract

An approach to the de novo structure prediction of proteins is described that relies on surface accessibility data from NMR paramagnetic relaxation enhancements by a soluble paramagnetic compound (sPRE). This method exploits the distance‐to‐surface information encoded in the sPRE data in the chemical shift‐based CS‐Rosetta de novo structure prediction framework to generate reliable structural models. For several proteins, it is demonstrated that surface accessibility data is an excellent measure of the correct protein fold in the early stages of the computational folding algorithm and significantly improves accuracy and convergence of the standard Rosetta structure prediction approach.

During the last few decades, NMR spectroscopy has become the method of choice for studying high‐resolution protein structures in solution. In the standard NMR‐based structure determination approach, structurally relevant data from different sources, such as pair‐wise interatomic distances and orientation information, are collected and used as restraints for structure calculation.^1^ Very recently, several groups have realized that the growing number of structural data available in the Protein Data Base^2^ (PDB) provide a valuable source for NMR‐based structure determination, in particular when combined with NMR chemical shifts.^3^ In these de novo structure prediction approaches, only the amino acid sequence is needed, and structures are calculated in an often Monte Carlo‐based conformation‐searching algorithm. The benefits of NMR chemical shift data in fragment selection and evaluation of structural quality have been recognized^4^ and impressively demonstrated.^3^, ^5^ However, this method is still limited to small proteins owing to computational bottlenecks^6^ and requires extensive sets of NMR‐based structural data, which are difficult to obtain in case of larger proteins as a result of the increasing complexity of NMR spectra and line broadening of NMR signals because of overall slower protein tumbling.

Herein we describe an approach in which we exploit NMR‐based surface accessibility data obtained from measurement of paramagnetic relaxation enhancements induced by a soluble paramagnetic compound for de novo structure prediction in the Rosetta framework.^6^, ^7^ The addition of soluble paramagnetic compounds leads to a concentration‐dependent increase of relaxation rates, the so‐called paramagnetic relaxation enhancement (here denoted as solvent PRE, sPRE; also known as co‐solute PRE, Figure 1 a). This effect depends on the distance of the spin to the protein surface, with the spins on the surface being affected most, and has been shown to correlate well with protein structure.^8^ sPREs have been exploited for structural studies of biomolecules such as for structure determination of proteins,8a, 9 docking of protein complexes,^10^ and detection of dynamics^11^ in the recent years.

**Figure 1:**
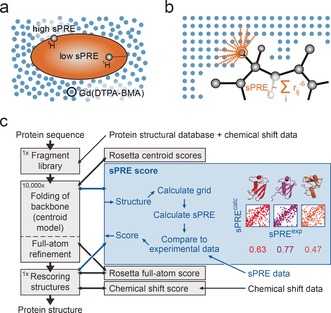
Principle of sPRE‐CS‐Rosetta. a) NMR sPRE data provides quantitative and residue specific information on the solvent accessibility as the effect of paramagnetic probes such as Gd(DTPA‐BMA) is distance dependent. b) Back‐calculation of sPRE data relies on placing the protein into equidistantly spaced grid points, while overlapping grid points are removed. The sPRE is approximated by the sum of all contributions of the surrounding grid points. c) The sPRE module is implemented as a scoring function capable of scoring centroid as well as full‐atom models. At its core, the experimental sPRE data (sPRE^exp^) is compared to the predicted sPRE data of the current Rosetta model (sPRE^calc^) and a score based on the Spearman correlation coefficient (colored numbers) is computed. In this scheme, the sPRE score is used during the folding of the protein backbone using the simplified centroid model as well as for rescoring the final full‐atom models.

Although sPRE data has been used to evaluate structural quality, its use in structure calculations has been limited owing to the lack of time‐efficient computational methods for back‐calculation of sPRE data. This is essential because in Rosetta, every scoring function (that is, the sPRE score) is evaluated several ten thousand times for obtaining a single structure. Furthermore, a typical structural ensemble required for accurate structure prediction contains at least several thousands of such structure models, emphasizing the need for efficient scoring functions. Recently, an approach has been presented for the molecular dynamics software XPLOR‐NIH using a structure‐based metric including the neighboring heavy atoms.^9^ Herein, we use a different approach optimized for high‐performance and time‐efficiency in which we directly use a model structure and map it onto a bit array (Figure 1 b). This simplifies the required computations to simple grid‐based operations that are further accelerated by lookup tables. In this approach, the protein is placed in a regularly spaced grid represented by a three‐dimensional bit array. Grid positions that overlap with the protein are marked, such that the remaining unmarked grid positions represent the inverted shape of the protein, and can be regarded as a spatial distribution of the paramagnetic agent. The sPRE of a protein atom is then calculated by summing up all contributions of the unmarked grid positions within the integration radius around the atom (Figure 1 b).

We then extended the Rosetta de novo structure prediction method to incorporate sPRE data to take advantage of the surface accessibility information in the folding of the protein backbone (Figure 1 c). A new scoring function for sPRE data was implemented and is available to the entire Rosetta framework. In short, the sPRE module first back‐calculates the sPRE data for a given structure using the grid‐based algorithm described above. The back‐calculated sPRE data is then compared to the experimental sPRE data using the Spearman correlation coefficient and converted into an energy score (sPRE score).

The suitability of sPRE‐based surface‐accessibility data as an indicator of structural accuracy was evaluated for the individual CS‐Rosetta refinement stages using a set of proteins ranging from of 6.4 to 41 kDa. To this end, we created structural ensembles for the individual stages of the Rosetta AbinitioRelax protocol and compared the sPRE score to the Rosetta scores. We observed that the sPRE score outperforms the initial scores in the early protein folding stage I, which has been initially optimized to collapse the extended chain but also in the later stages II–IV in which the fold of the backbone is determined (Figure 2 a; Supporting Information, Figure S1). Over a wide C^α^‐RMSD range of 3–20 Å, the sPRE score shows a clear correlation with structural accuracy. In the later stages II–IV, the quality of the standard Rosetta scores improves and they cooperate with the sPRE score when combined. This strongly indicates that the sPRE score is capable of guiding the sampling of a Rosetta AbinitioRelax run towards the native structure. Interestingly, for near‐native‐like structures (C^α^‐RMSD <2 Å), the Rosetta score shows a better performance compared to the sPRE score. This is probably due to the higher susceptibility of the sPRE to variations on the protein surface where minor conformational changes, for example, side‐chain rotations, translate into a large variation of the sPRE. Summarizing, our findings suggest that sPRE data can be valuable for Rosetta‐based de novo structure prediction when sampling states far from the native state, since it is able to guide it towards more native‐like states. From these more native‐like states, the common Rosetta scoring functions are able to drive the sampling to high‐resolution, full‐atom structures.

**Figure 2:**
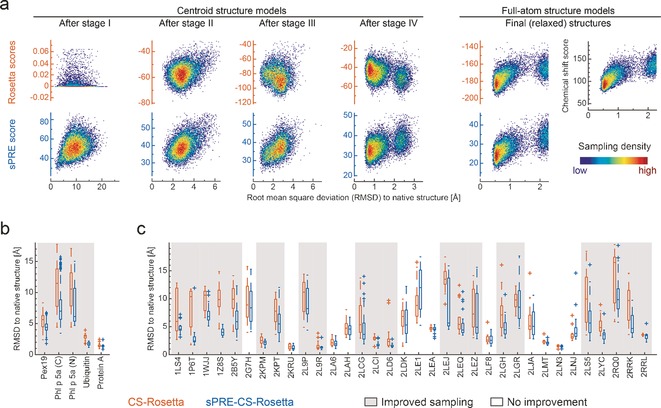
sPRE data is an excellent measure of the correct protein fold and improves protein structure prediction. a) Structural ensembles of ubiquitin representing different stages of the AbinitioRelax protocol were rescored using Rosetta centroid and full‐atom scores (orange axis), the sPRE score (blue axis), and the chemical shift score (black axis). Experimental sPRE data for H^N^ and H^aliphatic^ protons were used as input for the sPRE score. b), c) Box plots showing the average C^α^‐RMSD to the native structure for models obtained from CS‐Rosetta (orange) and sPRE‐CS‐Rosetta (blue). sPRE data was determined by NMR experiments (b) or back‐calculated (c). All obtained structural models were scored according to the sum of the Rosetta, chemical shift and sPRE score (b) or according to the sum of the Rosetta and the chemical shift score (c). For every protein, the best scored 0.2 % structures of all models were selected and used to generate the box plots. Proteins for which the sampling was improved by the sPRE module (reduced mean RMSD to native structure compared to CS‐Rosetta) are marked with a gray background and proteins for which CS‐Rosetta and sPRE‐CS‐Rosetta failed are not shown (average C^α^‐RMSD >10 Å in the case of p16, 1CX1, 1F2 H, 1GXE, 1IX5, 1ON4, 1RFL, 1XWE, 2KNR, 2LFC, 2LFP, 2LLL, 2PQE, 2RRF, 3ZQD, and 4A5V). All scores are shown in arbitrary units.

To examine the potential of solvent accessibility data for Rosetta de novo structure prediction, we carried out classical CS‐Rosetta as well as CS‐Rosetta with sPRE scoring (referred to as sPRE‐CS‐Rosetta) calculations with experimental NMR data (Figure 2 b; Supporting Information, Table S1) and back‐calculated sPRE data (Figure 2 c; Supporting Information, Table S2). For ubiquitin and using experimental amide (^1^H^N^) and aliphatic (^1^H^aliphatic^) proton sPRE data, the sPRE‐CS‐Rosetta approach improved the sampling significantly in a set of about 10 000 models (Figure 3). As a result, more structures in the C^α^‐RMSD range up to 1.5 Å were sampled, and subsequently the common Rosetta scores converge to high‐resolution structures as close as 0.7 Å C^α^‐RMSD to the native structure. The main structural difference of the ubiquitin ensemble at 2.5 Å compared to the ensemble at about 0.7 Å is a register shift of β‐strand 5 (Figure 3 a). To evaluate the robustness, we carried out sPRE‐CS‐Rosetta calculations using only subsets of the experimental sPRE data. Surprisingly, even with restricted sPRE data sets (^1^H^N^, sidechain ^1^H, or ^1^H^α^/^1^H^β^) the sampling was not deteriorated (Supporting Information, Figure S2 a). This suggests that the surface‐accessibility information is already encoded in a low number of sPRE restraints and that scoring the global fold of a protein does not require precise input data as long as the correct trend of the solvent accessibility pattern is present in the data. This is further supported by the observation that a complete set of synthetic ubiquitin sPRE data did not further improve the structural quality (Supporting Information, Figure S2 b). Summarizing, this indicates that even in case of sparse and incomplete chemical shift assignments, sPRE data can provide high‐quality structural models. Similar results were obtained using experimental data for the C‐terminal domain of Phl p 5a, a four helix bundle in which case the sPRE‐CS‐Rosetta approach significantly improved convergence and accuracy of the structural models in a set of about 100 000 models (Figure 3 b; Supporting Information, Figure S3).

**Figure 3:**
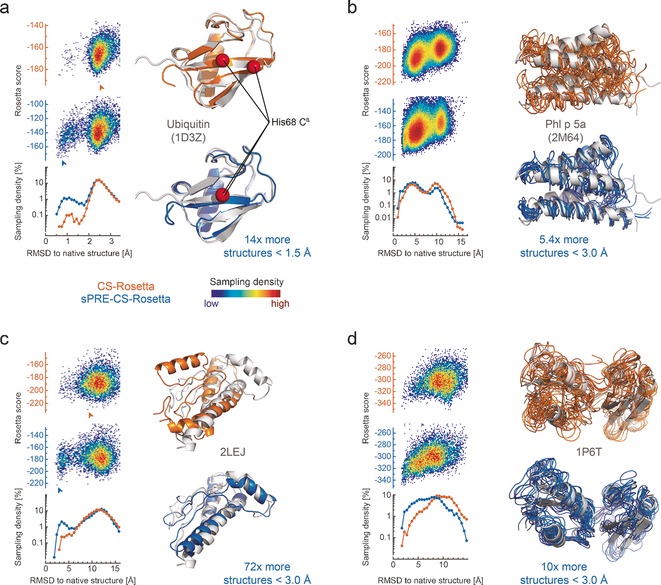
sPRE data enhances accuracy and convergence of CS‐Rosetta structure prediction. The lowest‐energy models of CS‐Rosetta (orange) and sPRE‐CS‐Rosetta (blue) are compared to the NMR solution structures (gray, PDB code). For both methods, the corresponding Rosetta score (score13_env_hb) is plotted on the left and the distribution of the C^α^‐RMSD of the sampled structures is shown below for both methods in a logarithmic histogram. For ubiquitin (a) and the C‐terminal domain of Phl p 5a (b) experimental sPRE data for amide and aliphatic protons is used, and for human prion protein (c) and the P‐type ATPase CopA (d) the input sPRE data was back‐calculated using the lowest energy model. In (a) and (c), the best scored model according to the Rosetta score is shown (see arrow in score plots), and for (b) and (d) the 10 lowest‐energy models are shown. For ubiquitin (a), a red sphere represents the position of the C^β^ atom of His 68, indicating the wrong positioning of the β‐strand in the CS‐Rosetta run. A more detailed picture of the scores is shown in the Supporting Information, Figure S3. All scores are shown in arbitrary units.

To further examine the potential of solvent accessibility data for Rosetta structure prediction, we built a benchmark of challenging proteins with sizes up to 170 residues and using synthetic sPRE data (Supporting Information, Table S2). The structure of each protein was subsequently determined using classical CS‐Rosetta as well as sPRE‐CS‐Rosetta. Comparing the average C^α^‐RMSD to the native structure for the best 0.2 % models, filtered by the sum of Rosetta and chemical shift score, revealed that for several proteins (22 of 49) the accuracy of the structure prediction was notably improved to models closer to the native structure (Figure 2 c). To solely account for the sampling improvement, we additionally filtered 1 % of the models that are closest to the native structure and compared the average C^α^‐RMSD of these sets (Supporting Information, Figure S4). These results show that for most of the tested proteins (30 of 49) the sampling is significantly improved. Two proteins of this benchmark, 2LEJ and 1P6T, are illustrated in Figure 3 c and 3 d, respectively. To further evaluate the robustness of the sPRE scoring module, we determined the structure of four proteins using back‐calculated sPRE data with an increasing level of noise, various assignment completeness and different sets of resonances (Supporting Information, Tables S3 a–d). Our results for fully assigned proteins and using only sPRE data for H^N^, H^α^, and H^β^ resonances show that the sampling is improved even in the presence of simulated noise with a range of four times the sPRE value (±2 sPRE value, here denoted as noise level of 200 %). Moreover, even for partially assigned proteins and using only amide protons, which corresponds to less than one restraint per residue, the number of models close to the native structure is still enhanced. Therefore, the results of the benchmark showed that solvent accessibility data improves accuracy and convergence even if only sparse data is available.

To further evaluate the performance of sPRE‐CS‐Rosetta in combination with (sparse) NMR‐based structural data, we carried out de novo structure predictions using random subsets of experimental nuclear Overhauser enhancement (NOE)‐based distance and residual dipolar coupling (RDC)‐based orientation data. Most notably, the addition of experimental sPRE data increases the sampling significantly in all cases (Supporting Information, Figure S5, Tables S4 a–b, S5). This confirms that the sPRE data acts as an orthogonal restraint.

Iterative sampling has been shown to improve Rosetta‐based de novo structure prediction in some cases. We compared the performance of our approach to the iterative sampling algorithm CS‐Rasrec‐Rosetta.^12^ We find that the performance of the Rasrec‐based structure predictions does not improve significantly in terms of sampling (that is, the RMSD of the best structures), but rather excludes the high‐RMSD structures during the iteration. In line with this, inclusion of sPRE data in the sPRE‐CS‐Rosetta shows significantly improved performance (Supporting Information, Figure S6). An explanation for the comparable performance of CS‐Rasrec‐Rosetta is the fact that the Abinitio part of the classical Rosetta is still an integral part of CS‐Rasrec‐Rosetta.

Our findings for several model proteins show that sPRE data improves conformational sampling and scoring of CS‐Rosetta, subsequently provides more accurate and better converged structural models, and thereby effectively shifts the size limitations of CS‐Rosetta. Our observation that a restricted set of sPRE data is sufficient to improve structural quality indicates that this class of restraints will be particularly powerful for de novo structure prediction of larger proteins where complete chemical shift assignments are difficult to obtain. With this respect sPRE data can be used in combination with (sparse) restraints from conventional approaches and offer several benefits over conventional approaches based on NOE‐derived distance restraints only: sPRE data can be obtained for any kind of NMR‐active nucleus for which chemical shift assignments are available (including for example ^13^C8a), and as long as a NMR spectrum can be obtained. This is independent of the completeness of chemical shift assignments which is essential for NOE‐based approaches. Combination of the sPRE‐CS‐Rosetta approach with recently developed iterative sampling algorithms,^12^ or comparative modeling^13^ in the future promises further improvements for de novo structure prediction of larger proteins. In these cases, surface accessibility data can be particularly useful as it provides orthogonal information compared to other NMR restraints that often contain local, short‐distance information. Furthermore, the sPRE module is open to complementary types of surface accessibility data such as for example bioinformatics and mass spectrometry (cross‐linking, radical‐mediated protein footprinting) data and will thereby allow integrating different techniques in one program.

## Supporting information

As a service to our authors and readers, this journal provides supporting information supplied by the authors. Such materials are peer reviewed and may be re‐organized for online delivery, but are not copy‐edited or typeset. Technical support issues arising from supporting information (other than missing files) should be addressed to the authors.

Supplementary
