# Supplementary material for: Prediction of Protein Structure Using Surface Accessibility Data
Source: Angew Chem Int Ed Engl. 2016 Aug 25;55(39):11970–4. doi: 10.1002/anie.201604788 (PMC5026166; doi:10.1002/anie.201604788)
Supplement: Supplementary file 1 — Supplementary [file ANIE-55-11970-s001.pdf]

Angewandte Chemie
Eine Zeitschrift der Gesellschaft Deutscher Chemiker

Supporting Information

Prediction of Protein Structure Using Surface Accessibility Data

*Christoph Hartlmüller, Christoph Göbl, and Tobias Madl**

anie_201604788_sm_miscellaneous_information.pdf

Contents

Experimental Section 1
Rosetta framework and the sPRE energy module. 1
Code availability 2
Input data 2
Back-calculation of sPRE data 2
Scoring a structural model 3
Verification of the sPRE back-calculation 4
Optimizing the sPRE module 4
Computational costs 5
Scoring Benchmark 5
Sampling Benchmark 6
Sampling Benchmark using NOE and RDC data 7
Comparison with RasRec 7
Protein expression and purification 7
NMR spectroscopy 8
Recording of sPRE data 8
Required measurement time 8
Measurement of sPRE data used in this study 9
Analysis of NMR data 9
Supplementary Figures 11
Supplementary Tables 20
References 31

Experimental Section

Rosetta framework and the sPRE energy module.

The sPRE module is capable of scoring full-atom models as well as simplified centroid models, which are used by CS-Rosetta during the folding of the backbone in the AbinitioRelax protocol. In this work, we used the classical CS-Rosetta protocol for structure determination consisting of i) a fragment selection using chemical shift data, ii) the AbinitioRelax protocol for generating structures and iii) a final rescoring of the structure ensemble.

It should also be noted, that the AbinitioRelax protocol is a combination of the Abinitio and the Relax protocol. The sPRE score is only used in the Abinitio folding algorithm but not in the Relax stage. The Relax algorithm is a gradient-based energy minimization step and is the last step of the AbinitioRelax protocol. At that late stage, the global protein fold is already determined by the Abinitio protocol and the Relax algorithm performs a full-atom refinement of the sidechains, without introducing extensive changes that could affect the overall fold of the protein. Therefore, the sPRE score is only used in the Abinitio stage where the global fold is determined but will not affect the final energy minimization. For the final rescoring of the full-atom structures, we used Rosetta's full-atom scores, the chemical shift score as well as the sPRE score. The Rosetta scoring functions as well as the chemical shift score of the Rosetta framework were used without any adjustments. As an input for the chemical shift score, we

use the published chemical shift data (the corresponding BMRB codes are listed in supplementary table 1).
Implementing a sPRE scoring function in the Rosetta framework, requires the back-calculation of sPRE data for a given protein model and the comparison of this back-calculated data with experimental data. The sPRE module is implemented as a WholeStructureEnergy and communicates with Rosetta using the common scoring interface of the framework. The module integrates seamlessly into existing Rosetta protocols and can easily be activated in every Rosetta application by assigning a non-zero weight to the identifier "spre" in the score function weighting set. Once the sPRE module is activated, an input file containing the sPRE data in talos file format (NMRPipe Table Format) has to be supplied. For details regarding code availability, setup instructions as well as a tutorial, please refer to http://mbbc.medunigraz.at/en/research/forschungseinheiten-und-gruppen/research-group-prof-madl/.

Code availability

In this work, we tested the sPRE module in conjunction with the AbinitioRelax and score_jd2 protocols of Rosetta 3.2 for structure calculation and rescoring structural ensembles, respectively. The sPRE module is entirely included in the Rosetta framework, requires no additional software or online service and will be made available in the upcoming releases of the Rosetta framework.

Input data

Although the module was mainly tested with proton sPRE data as input, the module supports input data for all carbon and proton atoms. The input data is adjusted as follows. For methyl groups, the sPRE values of all protons and the corresponding center carbon atom (if sPRE data is available) are averaged and assigned to the hetero atom. Similar re-mapping is done for tyrosine and phenylalanine sidechains in which case the protons and carbon atoms at the meta positions are averaged with the para carbon atom and mapped onto the para carbon atoms. For data sets that were not assigned stereo-specifically (non-stereo specific assignment is assumed by default), the sPRE values of prochiral protons are averaged with the corresponding carbon atom and projected onto the center atom (i.e. for serine, data for both H^{β} protons and for the C^{β} carbon atom is averaged and assigned to the C^{β} atom). Missing data for carbons or protons does not affect the averaging. As the paramagnetic effect shows a γ^{-2} dependency, where γ is the gyromagnetic ratio of the corresponding nucleus, all sPRE values are normalized by γ^{2} to allow proper averaging of sPRE data of different types of nuclei.
To score centroid models, further mapping needs to be performed since centroid models contain only 7 atoms per residue (H, N, C^{α}, C, O, C^{β} and a sidechain pseudo atom CEN). Consequently, the data for H^{α} is averaged with data for C^{α} (if available) and assigned to C^{α}. In a similar manner, data for H^{β} is assigned to C^{β} and all other sidechain data is merged and assigned to the CEN atom.

Back-calculation of sPRE data

sPRE data for a given structural model is back-calculated by an optimized grid-based approach (compare figure 1b) similar as described in previous studies.[1] In a first step, a uniform grid with a typical spacing of 0.5-2 Å between grid points (default 2 Å) is created around the given structural model. By default, every atom of the protein is at least 10 Å away from the boundaries of the grid.
Next, the atom positions of the protein are discretized onto this grid by replacing the x, y and z coordinates of the atom with the coordinates of the closest grid point. In a third step, grid positions that fall within the van-der-Waals region of the protein are marked as occupied. The atom radius is obtained from the Rosetta database. For centroid pseudo atoms, the radius is approximated by the distance between the centroid atom and the C^{α} atom of the same amino acid. All atom radii are increased by the radius of the paramagnetic agent (default 3.5 Å[1b, c]). This effectively marks all positions of the grid that are not accessible by a paramagnetic agent.
Next, the sPRE value for every atom is approximated by the sum of all grid positions within an integration radius (default 10 Å) that have not been marked as occupied in the previous step:

$$\mathrm{sPRE}_i^{\mathrm{model}} = \sum_{r_{i,j} < d_{\mathrm{int}}}^{N} \frac{1}{{r_{i,j}}^6} \cdot m_j \tag{1}$$

where i is the index of the protein atom, j is the index of the grid point, N is the number of grid points, $\mathrm{sPRE}_i^{\mathrm{model}}$ is the approximated sPRE value for the ith atom of the given protein structure, $r_{i,j}$ is the

discretized distance on the grid between the ith atom and the jth grid position, d_{int} is the integration radius (default 10 Å), and $m_j = \begin{cases} 0 & \text{if } j\text{-th grid point marked as occupied} \\ 1 & \text{else} \end{cases}$. Since the protein atoms and the grid positions are discretized on the same grid, $\frac{1}{r_{i,j}{}^6}$ is computed beforehand and stored in a lookup table.

Scoring a structural model

After back-calculating the sPRE data for every atom of the protein, the calculated values $\text{sPRE}_i^{\text{model}}$ are compared to the experimental data $\text{sPRE}_i^{\text{exp}}$. By default, the sPRE module uses a robust spearman correlation coefficient to compute a scalar score. To calculate the spearman correlation coefficient, both data sets ($\text{sPRE}_i^{\text{model}}$ and $\text{sPRE}_i^{\text{exp}}$) are ranked independently, generating two new data sets $\text{r}_i^{\text{model}} \in [1, \text{n}]$ and $\text{r}_i^{\text{exp}} \in [1, \text{n}]$. The raw score is then obtained using

$$s_{\text{spearman}} = 1.906 \cdot (1 - \text{correlation}_{\text{spearman}}) - 0.144$$

$$= 1.906 \cdot \left(1 - \frac{\sum_{i=1}^{\text{n}} \left(\left(\text{r}_i^{\text{model}} - \overline{\text{r}^{\text{model}}} \right) \cdot \left(\text{r}_i^{\text{exp}} - \overline{\text{r}^{\text{exp}}} \right) \right)}{\sqrt{\sum_{i=1}^{\text{n}} \left(\text{r}_i^{\text{model}} - \overline{\text{r}^{\text{model}}} \right)^2 \cdot \sum_{i=1}^{\text{n}} \left(\text{r}_i^{\text{exp}} - \overline{\text{r}^{\text{exp}}} \right)^2}} \right) - 0.144 \qquad (2)$$

where $\text{correlation}_{\text{spearman}}$ is the correlation coefficient of the ranked data sets $\text{r}_i^{\text{model}}$ and r_i^{exp}, i is the index of a protein atom for which experimental sPRE data is available, n is the number of atoms for which experimental sPRE data is available, $\text{r}_i^{\text{model}}$ is the ranked back-calculated sPRE data of the ith atom, r_i^{exp} is the ranked experimental sPRE data of the ith atom and $\overline{\text{r}^{\text{model}}} = \frac{1}{\text{n}} \sum_{i=1}^{\text{n}} \text{r}_i^{\text{model}}$ as well as $\overline{\text{r}^{\text{exp}}} = \frac{1}{\text{n}} \sum_{i=1}^{\text{n}} \text{r}_i^{\text{exp}}$ correspond to the average ranks over all atoms. The scaling of 1.906 and offset of 0.144 were chosen such that for a test set of small proteins (see supplementary table 6) the raw score of most final full-atom structures ranges from 0 to 1.
The spearman correlation was chosen as the default method from a set of several alternative methods. In this study, several other scores were tested and all of these scores were computed according to equation (3)

$$s_{\text{score}_j} = A_j \cdot \tilde{s}_{\text{score}_j} - B_j \qquad (3)$$

where $\tilde{s}_{\text{score}_j}$ denotes the raw score, s_{score_j} is the scaled score, and A_j as well as B_j are constants. s_{score_j} was then normalized with an appropriate power of the average reference sPRE. For every type of score, the values for A_j and B_j as well as the computation of $\tilde{s}_{\text{score}_j}$ are listed in the supplementary table 7.

The scores based on the Pearson correlation (s_{pearson}), Spearman correlation (s_{spearman}) and the quadrant count ratio (s_{quadrant}) are derived from the corresponding correlation coefficients and as a consequence are mathematically bounded. All other scores in the supplementary table 7 are unbounded and normalization of these scores becomes more challenging. Here, we used a test of fully-relaxed, full-atom protein structures for normalization. As structure models in the initial phase of CS-Rosetta are entirely different compared to the optimized final models, the chosen set of constants A_i and B_i can give rise to large score values for these initial structural models. Furthermore, the absolute values of these scores depend on the size of the protein as well as the number of total constraints and can be dominated by outliers. Therefore, we only considered s_{pearson}, s_{spearman} and s_{quadrant} for optimizing the sPRE module, since those scores are based on correlation coefficients and resulted in a stable and robust folding algorithm. Moreover, the correlation-based scores can be utilized in scenarios involving different proteins, different sets of sPRE data or a mix of centroid and full-atom models.
In addition to the fixed A_i and B_i values, the score is scaled and shifted before it is returned to the Rosetta framework. This scaling step can be adjusted by the user and is performed according to

$$\text{sPRE score} = \text{scaling} \cdot s_{\text{score}_i} - \text{offset} \qquad (4)$$

where s_{score_i} is the score as calculated above, scaling is given by the Rosetta command line option -score:spre:scaling (default 67), and offset is given by the option -score:spre:offset (default 0).

Note that the sPRE score (as any other scoring function used in Rosetta) is also scaled according to the Rosetta weight sets. The Rosetta weight for the sPRE score was set to 1.0 throughout the study. Furthermore, it should be noted that the Monte-Carlo algorithm of Abinitio relies on differences between scores. The offset was implemented only for the sake of completeness and chosen to be 0.

Verification of the sPRE back-calculation

The sPRE module uses a discretized, low resolution back-calculation of the sPRE data. To visualize the error of this approximated back-calculation, we compared the back-calculated sPRE data obtained from the Rosetta sPRE with data obtained from a classical grid-based back-calculation (Supplementary figure 7). As expected, the low resolution of the grid results in a weakening of the correlation. However, even with a grid of 2 Å, solvent exposed residues are still predicted to have a high sPRE, indicating that such low-resolution back-calculated data can still be used to guide the Rosetta sampling algorithm towards the native structure. Furthermore, assuming a global protein, the accuracy of the approximated sPRE back-calculation increases as the size of protein increases. For larger proteins, the effect of missing some high resolution structural features can be neglected compared to the large sPRE gradient between the core and the surface of the protein structure.

Optimizing the sPRE module

The sPRE scoring function can be directly adjusted using several parameters most notably, the resolution of the grid, the cut-off radius (integration radius) and the method for comparing the measured and the back-calculated sPRE data. An example showing how these parameters affect the scoring performance is illustrated in supplementary figure 8. In cases where the sPRE score is used in the Abinitio protocol of CS-Rosetta, two additional parameters become crucial, the global weight as well as a stage-specific weighting of the sPRE score. We optimized these parameters using a set of proteins (Supplementary table 6) and the recommended settings are listed in supplementary table 8.
The optimal values for the grid resolution and the cut-off radius can vary depending on the size of the protein, the quality of the sPRE data and the computational resources. Since both parameters affect the accuracy of the back-calculation as well as the computational costs, the optimal value is a trade-off between computational time and accuracy (Improving the resolution by lowering the distances between the grid points or enlarging the integration radius leads to a cubic increase of computational costs. For details see the section Computational costs).
As a rule of thumb, a minimum value of 10 Å is required for the integrational cut-off radius since smaller values lead to a significantly reduced correlation between the sPRE score and the C^{α}-RMSD to the native structure (Supplementary figure 8a). Increasing the integration threshold to more than 10 Å might be beneficial in the case of large proteins. However, given the current size limitations of CS-Rosetta, an integration threshold of 10 Å was sufficient throughout this work. Regarding the resolution of the grid, a grid spacing of 2 Å is sufficient in most cases and allows a fast computation of the sPRE score. Spacings above 2 Å lead to a significantly increased error and a broadening of the scoring correlation (Supplementary figure 8b). Higher resolved grids with a spacing of 1 Å or 0.5 Å increase the scoring performance in case where the score is used to distinguish between similar conformations with different high-resolution features (for example the tilting of the 4 helices in the case of C-terminal phpl5a, see figure 2d). In cases where the sPRE score guides the folding mainly in the early folding stages, an increase in resolution is typically not beneficial but requires more computational resources. In summary, using a grid spacing of 2 Å and an integration of 10 Å is a good compromise between performance and accuracy for most cases. To include high resolution information for near native-like structures, a grid spacing of 0.5 to 1 Å is preferable.
To find the optimal method of comparison, as well as the optimal global and stage-specific weights we used the following approach. We first chose a set of small to medium-sized proteins (Supplementary table 6) and predicted structure models using classical CS-Rosetta and sPRE-CS-Rosetta with different settings of the sPRE score. We then computed the percentage of models close to the experimental NMR structure (below 4 Å for 1Q02, 2JTV, 2JMB and 2CKX, below 1.5 Å for 2OSQ and 2K52) and used it as a measure for convergence for every parameter value. Using this procedure to optimize the global weighting, we found an optimum for the global scaling factor of 67 in our test set (Supplementary table 9). Although this default choice resembles a good compromise for many scenarios, it should be noted that the optimal scaling factor varies between proteins. Over-emphasizing the sPRE score can lead to physically incorrect structures, while a low weighted sPRE score fails to drive the sampling in a significant manner. The scaling of the sPRE score can therefore be adjusted by the user either by changing the score weight set within of the Rosetta framework or by changing the scaling command line option of equation (4).

We then used the same strategy to evaluate how different weightings in the individual Abinitio stages affect the sampling. In our test set, we found that every stage of the Abinitio protocol can benefit from the sPRE score (Supplementary table 10). Furthermore, depending on the protein, the improvement of the sampling can be traced back to different stages of the Abinitio protocol. Therefore, our tests suggest to include the sPRE score in all stages of Abinitio with a constant weight throughout the protocol.
Among the five stages of Abinitio, stage I uses the simplest scoring function, solely based on a van-der-Waals term. We still chose to include the sPRE score in this early stage, as the sPRE score depends on the global fold and favors compact structures. It is therefore suited to collapse the initial extended chain which is the main purpose of stage I.
Eventually we tested different algorithms to compare the experimental and the synthetic data using the same procedure. We found different types of correlation coefficients to perform best and to be the most robust among several common choices such as RMSD, correlation coefficient and Chi values (Supplementary table 7). Chi values and several variations of RMSD performed well only in a few test cases. The classical Pearson correlation coefficient performed considerably better, but was out-performed by the Spearman correlation coefficient which gave the best convergence in most cases (Supplementary table 11). Consequently, we chose the Spearman correlation as the default method to compare experimental and back-calculated sPRE data.
The Spearman correlation coefficient is obtained by ranking both data sets (measured and back-calculated sPRE data) independently, and subsequently calculating a classical Pearson correlation coefficient of the ranks. As the ranks are bounded by the number of data points, the Spearman correlation is robust and less sensitive for outliers, making it well suited to cope with amide proton sPRE data that might contain additional relaxation contribution due to chemical exchange with water. Also note that due to the ranking of the input data, the sPRE module can potentially be used to include solvent accessibility data from different sources such as bioinformatics or other experimental methods such as mass spectrometry.

Computational costs

The main contribution of the overall computational effort is the back-calculation of the sPRE data. We therefore approximated the sPRE by discretizing the protein atom positions to positions of the same grid that is used to model the paramagnetic substance (see chapter Back-calculation of sPRE data). This simplifies the required computations to simple grid-based operations that can be accelerated by techniques such as lookup tables. We also aimed to reduce the total amount of memory to improve cache efficiency.
To quantify the computational costs of our sPRE score, we compared the runtime of rescoring an ensemble of ubiquitin structural models using the sPRE score with the runtime of computing the Rosetta centroid scores. As shown in supplementary table 12, the computational costs mainly depend on the resolution of the grid and the radius of integration. As an example, choosing a grid resolution of 1 or 2 Å requires an extra computational cost that is in the same order as required by the efficient centroid Rosetta scores (about 80% more computational time compared to only calculating a Rosetta centroid score). Furthermore, we compared the computational costs of CS-Rosetta and sPRE-CS-Rosetta (Supplementary table 13). The extra costs of the sPRE module do not change the order of magnitude of the total runtime when choosing a grid with 2 Å spacing (computational cost roughly doubles). Reducing the grid to 1 Å requires about 5 to 6 times the computational time compared to a classical CS-Rosetta run. It should be noted that in this comparison, the number of computed structures was kept constant. In practice, using the sPRE score can dramatically speed up the complete procedure, as less models need to be computed to sample near-native conformations. The additional computational costs of the sPRE module become even less important considering that CS-Rosetta can easily by parallelized and the number of computational cores in modern clusters increases rapidly.

Scoring Benchmark

To evaluate the potential of the sPRE score, we performed a comparison between the common Rosetta scoring functions and the sPRE score. In particular, we did not limit the benchmark to fully-relaxed full-atom structures, but we also analyzed how the sPRE score performs in the case of centroid structure models since those simplified models are used to fold the extended chain in the Abinitio protocol.
In a first step, we chose a set of proteins for which the native structure was determined by NMR spectroscopy and experimental sPRE data was either measured or already available in (Supplementary table 1). For every protein, a test ensemble of structures was generated by starting classical CS-Rosetta structure prediction runs and collecting the centroid models at the end of each stage (stage I, II, III and IV) as well as the final full-atom structures.

To ensure that the ensemble covers a broad RMSD range, from only partially-folded proteins to near native structures, we added distance restraints that were derived from the native structure. Gradually improved structures were obtained by running several CS-Rosetta runs and narrowing the distance potential in steps of 10, 6, 4, 3, and 2 Å. In total, we generated 15000 models for every protein and for every stage (3000 per protein, Abinitio stage and distance potential window size).
These ensembles were then scored using the corresponding centroid Rosetta score (score0 for stage I, score1 for stage II, score2 for stage III and score3 for stage IV) and the sPRE score. The chemical shift score was only used for fully-relaxed structures, as the score is only applicable to full-atom models.
The results of the scoring benchmark clearly suggest that the sPRE score can be used to find near-native structures (Supplementary figure 1). In particular in the case of centroid models, the Rosetta scoring function in some cases prefers wrongly folded models over near-native structures. For these cases, we observed that the sPRE score outperforms the Rosetta score (see for example supplementary figure 1a and b). On the other hand, for full-atom models the Rosetta and in particular the chemical shift score are more reliable and the performance of these scores is similar to that of the sPRE score. Although the sPRE score mainly depends on the global fold properties with only minor contributions from local high-resolution structural features, in some cases the sPRE score performs as well as the chemical shift score and outperforms the Rosetta full-atom score even in the low RMSD range (see for example supplementary figure 1c, g and h). Moreover, considering that in some cases only sPRE data for amide protons was used, it is interesting to note that in our test set we never observed the sPRE score to perform worse than the Rosetta centroid score.
In summary, the scoring benchmark revealed the potential of the sPRE score in finding native-like structures and consequently suggests the score to be perfectly suited to improve sampling and thus the overall performance of CS-Rosetta.

Sampling Benchmark

To study the benefit of including solvent accessibility data into the folding algorithm of CS-Rosetta, we built a test set of 49 proteins by randomly selecting protein models of the protein data base[2] (PDB) with a protein core size up to 170 residues and for which chemical shift data is available (Supplementary table 2). A full set of synthetic carbon and proton sPRE data was back-calculated using the lowest-energy model of the submitted structure in the PDB. We then predicted models using classical CS-Rosetta as well as sPRE-CS-Rosetta. For both methods, the obtained structure ensembles were ranked according to the sum of chemical shift score and Rosetta full-atom score (score13_env_hb) and the average C^{α}-RMSD of the best ranked 0.2% was computed (Figure 2c). To solely address the sampling of both methods, we also compared the best 1% by C^{α}-RMSD (Supplementary figure 4b). Proteins, for which both methods fail (average C^{α}-RMSD > 10 Å) where not analyzed. As indicated by the scoring benchmark, the additional solvent accessibility data significantly improved the sampling compared to classical CS-Rosetta.

To evaluate if the observed benefit is also present when using experimental NMR data, we repeated the sampling benchmark using a set of proteins for which experimental sPRE data is available (Supplementary table 1). We again predicted models using both methods and computed the average C^{α}-RMSD of the best ranked models by the sum of the chemical shift score, the Rosetta full-atom score (score13_env_hb) and the sPRE score (Figure 2b) as well as by the C^{α}-RMSD (Supplementary figure 4a). Although both methods failed to predict reasonable folds in the case of MBP and p16, the experimental sPRE data significantly improved sampling in the case of Pex19, Ubiquitin and both domains of Phl p 5a. In the case of Protein A, both methods resulted in high-resolution models.

Since the previous sampling benchmarks clearly showed that sPRE data improves the convergence and accuracy of CS-Rosetta, we used 4 proteins (2LEJ, 1LS4, 1P6T and 1Z8S) to quantify the robustness and applicability of sPRE-CS-Rosetta regarding typical challenges in protein NMR spectroscopy. For this benchmark, we first back-calculated a full sPRE dataset as described before. We then generated different sets of sPRE data by simulating incomplete assignments (40%, 70% and 100% assigned), different noise levels (30%, 60%, 100%, 200% and 400%) and different atom subsets (H^N only, H^N and $H^{methyl\text{-}ILV}$ as well as H^N, H^{α} and H^{β}). For a comparison of the simulated noise with experimental sPRE data see supplementary figure 9. Next, structure ensembles were predicted using sPRE-CS-Rosetta for every sPRE data set and the percentages of models with an C^{α}-RMSD of 5 Å or less to the native structure were computed for every ensemble as well as for the reference CS-Rosetta ensemble (Supplementary tables 3a-d). Interestingly, this sampling benchmark revealed the robustness of the sPRE score and clearly suggests its applicability to sparse and erroneous experimental NMR sPRE data.

Sampling Benchmark using NOE and RDC data

To show the orthogonality of the sPRE score with other experimental NMR data, the structure of ubiquitin was predicted using CS-Rosetta and sPRE-CS-Rosetta in the absence and presence of additional NMR restraints such as RDCs and NOEs (see supplementary table 4a-b and 5 as well as supplementary figure 5).

To this end, experiment NOEs and RDCs for ubiquitin (1 set of H^N-N RDCs recorded in one medium) were obtained from the literature (PDB entry 1D3Z). Next, random subsets of either NOE or RDC data were generated with a varying number of total restrains in the sets (see supplementary table 4a and 5). Ambiguous NOE restraints were counted as a single restraint and the AmbiguousRestraint groups of the Rosetta framework were used to account for the ambiguity. For every RDC subset, the CS-Rosetta toolbox (http://csrosetta.chemistry.ucsc.edu/) was used to prepare the RDC data for the usage in the Rosetta framework.

For every NOE or RDC subset, CS-Rosetta and sPRE-CS-Rosetta runs were used to obtain ensembles of ubiquitin with 5000 models each. For every subset size, 2 to 4 different random subsets were generated and used as input to account for random effects of the selection process (see supplementary tables 4a and 5). To compare the performance of the different input sets, the percentage of models with C^α-RMSD of 1.0 Å or less was computed for every ensemble. As the results show, the sPRE module improves the sampling of CS-Rosetta in all cases, even when using large sets of NOEs restraints.

In addition, the percentages of wrong models (C^α-RMSD above 4.0 Å) were analyzed using the same data set (see supplementary table 4b). The results show that adding the NOE scoring functions which consist of several thousands of NOEs not only generates more high-resolution structures, but also increases the percentage of models far from the native structure. This can be explained by the fact that such a NOE score containing a large number of restraints can become rather complex and therefore harder to sample efficiently. On the other hand, the sPRE score depends on the global fold and as such is less prone to rapid change upon minor conformational changes. With a smoother energy landscape, the sPRE score can in particularly drive the sampling from far off models to near native-like models. This can be seen by a reduction of models with a C^α-RMSD of 4 Å or more.

Comparison with RasRec

To compare the performance of CS-Rosetta and sPRE-CS-Rosetta to the iterative Rosetta protocol RasRec,[3] the Rosetta toolbox (http://csrosetta.chemistry.ucsc.edu/) was used to setup RasRec-Rosetta runs. The corresponding amino acid sequence as well as the chemical shift data as listed in supplementary table 1 were used as input data for the RasRec runs. The pool size of the RasRec protocol was increased to 1000 while all other settings were left as default. The obtained full-atom models were rescored using the same procedure as for ensembles obtained with CS-Rosetta and sPRE-CS-Rosetta. The results are compared in supplementary figure 6.

Protein expression and purification

For the expression of protein A and p16, a pET-M11 vector was modified to express the protein A as a solubility tag for p16 expression. The vector contains an N-terminal hexa-histidine sequence followed by protein A, a TEV (tobacco etch virus) cleavage site and the *E. coli* codon optimized DNA sequence of human p16. After cleavage by TEV protease, the protein A domain includes 16 N-terminal residues (MKHHHHHHPMKQHDEA) and an unstructured C-terminal region of 15 residues including the remaining cleaved TEV-site (MDAGSGSGSENLYFQ). The cleaved p16 protein contains two additional N-terminal residues (GA) followed by its 156 amino acids (canonical sequence, isoform 1). The expression vector was transformed into *E. Coli Bl21 (DE3)* and cells were grown at 37 °C, using 50µg/ml of kanamycin for selection. After inoculation of 150 ml of M9 minimal medium including uniformly ^{13}C labeled glucose (3 g per liter) and uniformly ^{15}N labeled ammonium chloride (1 g per liter), the culture was grown over night while vigorous shaking. In the next morning, the cell suspension was diluted with 850 ml of the same medium and grown to an OD of 0.8 and protein synthesis was induced by addition of IPTG (isopropyl-1-thio-D-galactopyranoside) to a final concentration of 0.5 mM. Then the culture was incubated over night at 19 °C and harvested on the next day. The cell pellets were re-suspended in 30 ml purification buffer (8 M urea, 20 mM TRIS, pH 8.0 and 20 mM Imidazole) and frozen at -20 °C. For purification of the protein, the cell pellet was thawed at room temperature, sonicated and applied to a Ni-NTA agarose (Qiagen) gravity column following the manufacturer's instruction. The gravity column with the bound protein was then washed with 50 ml urea buffer. Afterwards, the buffer was exchanged to HEPES buffer (110 mM potassium acetate, 20 mM HEPES (4-(2-hydroxyethyl)-1-piperazineethanesulfonic acid), pH 8.0, 2 mM β-mercaptoethanol (BME), 5% (v/v) glycerol and 20 mM

imidazole) by washing of the column with 20 ml. The protein was then eluted with HEPES buffer including 250 mM imidazole and concentrated to 5 ml in a centrifugal filter unit (Amicon Ultra-15 (Millipore, 3kDa molecular weight cut-off) and applied to size exclusion chromatography. After loading on a HiLoad 16/600 Superdex 75 pg (GE Healthcare Life Sciences, 50 mM sodium phosphate, 500 mM NaCl, 2 mM BME, pH 6.0) the target-protein containing fractions were pooled. The sample was dialyzed over night at 4°C against HEPES buffer using a 2 kDa MWCO ZelluTrans V series membrane (Carl Roth) after addition of 400 µl of a 0.1 mg/ml 6xHistidine tagged TEV protease solution. Next day, the solution was applied again to a Ni-NTA agarose column to separate the cleaved p16 while the TEV protease, traces of uncleaved protein and the protein A remained bound to the column. The p16 flow through fraction was buffer exchanged into a HEPES buffer (4 mM HEPES, pH 7.5, 5 mM DTT) by using a 5ml highTrap desalting column (GE Healthcare Life Sciences) and the final concentration for NMR measurements was 150 µM (including 10% D_2O). The protein A fraction was again eluted by HEPES buffer containing 250 mM imidazole and concentrated to 5 ml and a second size exclusion step was performed as described above which allowed the separation of the pure protein. Protein A was concentrated to 500 µl and buffer exchanged into NMR buffer (20 mM potassium phosphate buffer at pH 6.5, 50 mM NaCl) using the desalting column. The protein concentration of the final NMR sample (containing 10% D_2O) was 1 mM.

Expression, purification and assignment of Phl p 5a and Pex19 have been performed using standard methods and are described elsewhere (C. G., Margarete Focke-Tejkl, Evelyne Schrank, T. M., Simone Kosol, Christoph Madritsch, Nazanin Najafi, Sabine Flicker, Rudolf Valenta, Klaus Zangger, Nico Tjandra, *manuscript submitted*; Leonidas Emmanouilidis, Ulrike Schütz, Konstantinos Tripsianes, T. M., Juliane Radke, Robert Rucktäschel, Matthias Wilmanns, Wolfgang Schliebs, Ralf Erdmann, Michael Sattler, *manuscript submitted*). The chemical shifts have been deposited in the biological magnetic resonance data base (BMRB[4], accession code 19107) and NMR samples were prepared in 50 mM KPi buffer, 20 mM NaCl at pH 6.2.

Expression, purification and assignment of MBP and Ubiquitin has been previously described.[1b]

NMR spectroscopy

Recording of sPRE data

To obtain sPRE data by NMR spectroscopy, we used a saturation-based approach as described previously.[1b] Briefly, the R1 relaxation rates are determined by a saturation-recovery scheme followed by a read-out experiment such as a $^{1}H,^{15}N$ HSQC, $^{1}H,^{13}C$ HSQC or a 3D CBCA(CO)NH experiment. For proton saturation, a 7.5 ms ^{1}H trim pulse followed by a gradient was applied. Then, z-magnetization is build up during the recovery delay, ranging between several milliseconds up to several seconds. Iterating through the different recovery delays is done in an interleaved manner, and short and long delays were ordered in an alternating fashion. For every R1 measurement at least 8 delay times were recorded and for error estimation, at least one delay time was recorded as a duplicate.

The measurement of R1 rates was repeated for increasing concentrations of the relaxation-enhancing Omniscan and the sPRE was obtained as the average change of the proton R1 rate per concentration of the paramagnetic agent. After every addition of Omniscan, the recovery delays were shortened such that for the longest delay all NMR signals were still sufficiently recovered. The interscan delay was set to 50 ms, as the saturation-recovery scheme does not rely on an equilibrium z-magnetization at the start of each scan. All NMR samples contained 10% $^{2}H_2O$. Spectra were processed using NMRPipe[5] and analyzed with the NMRView[6] and CcpNmr Analysis[7] software packages.

Required measurement time

To record a full set of sPRE data for H^N and $H^{aliphatic}$ protons about 2 to 5 days of measurement time is required when using $^{1}H,^{15}N$ and $^{1}H,^{13}C$ HSQC-based pseudo-3D relaxation experiments. Acquiring relaxation rates for 4 to 6 different concentrations of the paramagnetic agent is sufficient for most proteins.

For example, using a 400 µM sample of p16 (16.6 kDa) and a 750 MHz magnet equipped with a TXI probe head (Bruker), one set of relaxation rates was measured in 7 to 8 hours (3.5 hours for a pseudo-3D $^{1}H,^{15}N$ HSQC with 8 scans,100 complex points and 12 exponentially-spaced delay points as well as 3 hours for a pseudo-3D $^{1}H,^{13}C$ HSQC with 4 scans, 175 complex points and 12 exponentially-spaced delay points). Using more scans for overnight experiments, relaxation rates for 6 different concentrations of the paramagnetic agent can be acquired in 3 days.

The total measurement time of sPRE data for a 300 µM sample of Phl p 5a (24.1kDa) using pseudo-3D $^{1}H,^{13}C$ HSQC required 22 hours of measurement time. The data was acquired on an Avance III Bruker

700 MHz NMR spectrometer using 4 scans, 128 complex points and 12 exponentially-spaced delay points for 5 different concentrations of the paramagnetic agent.

Measurement of sPRE data used in this study

Details of the assignment and acquisition of sPRE data for MBP and Ubiquitin were published previously[1b] and sPRE data of Pex19 was obtained according to the same protocol.

The assignment of protein A was achieved by transferring the published chemical shift data of BMRB entry 4023[8] and confirmation of the resonance positions by acquiring HNCO, HNCACO and HNCACB experiments. sPRE data of a uniformly ^{13}C, ^{15}N labeled 1 mM sample of the Z domain of protein A was recorded on a 600 MHz magnet (Oxford Instruments) equipped with an AV III console and cryo TCI probe head (Bruker). R1 rates of H^{α} and H^{β} were measured using CBCA(CO)NH read-out spectra at 25 °C in the presence of 0, 0.5, 1, 2, 5 and 10 mM Omniscan (GE Healthcare, Vienna, Austria).

For the assignment of p16, previously reported chemical shifts of p16[9] were obtained from the BMRB[4] (accession code 4086) and the assignment was confirmed by recording backbone HNCA as well as sidechain (H)CCH and H(C)CH tocsy spectra of uniformly ^{13}C, ^{15}N labeled p16 on a Avance III Bruker 900 MHz NMR spectrometer at 25 °C. sPRE data of a uniformly ^{13}C, ^{15}N labeled 400 µM sample of p16 was recorded on a 750 MHz magnet (Bruker) equipped with an AV III console and TXI probe head (Bruker). R1 rates of aliphatic protons and amide protons were measured using ^{1}H,^{13}C HSQC and ^{1}H,^{15}N HSQC read-out spectra, respectively, at 25 °C in the presence of 0, 1, 1.75, 2.5, 3.25, 4 and 4.75 mM Omniscan (GE Healthcare, Vienna, Austria).

sPRE data of uniformly ^{13}C, ^{15}N labeled 0.3 mM Phl p 5a was recorded on an Avance III Bruker 700 MHz NMR spectrometer at 24.8 °C in the absence and after addition of 1, 2, 3 and 5 mM Omniscan (Nycomed, Oslo, Norway). R1 rates of aliphatic protons were measured using ^{1}H,^{13}C HSQC read-out spectra and amide R1 rate were obtained using ^{1}H,^{15}N HSQC read-out spectra.

Analysis of NMR data

Analysis of sPRE data for MBP and Ubiquitin was described previously[1b] and sPRE data for Pex19 was analyzed accordingly. For p16, Phl p 5a and protein A, the sPRE data was analyzed as follows. Peak intensities were extracted using the nmrglue[10] Python package and fitted to a mono-exponential build up curve using the SciPy python package and equation (5)

$$\mathrm{I}(t) = -A \cdot e^{-R_1 \cdot t} + C \qquad (5)$$

where $\mathrm{I}(t)$ is the peak intensity of the saturation-recovery experiment, t is the recovery delay, A is the amplitude of the z-magnetization build-up, C is the plateau of the curve and R_1 is the longitudinal relaxation rate. To estimate the error for the fitted rates R_1, the experimental error was estimated using duplicate recovery delays. For every R1 experiment, one absolute error for all peaks $\varepsilon_{\mathrm{exp}}$ was obtained by equation (6)

$$\varepsilon_{\mathrm{exp}} = \sqrt{\frac{1}{2N} \cdot \sum_{i=1}^{N} \delta_i} \qquad (6)$$

where N is the number of peaks in the spectrum, i is the index of the peak, and δ_i is the difference of the duplicates for the i-th peak. The error of the rates R_1 was then obtained using a Monte Carlo-type resampling strategy. By randomly drawing $3 \cdot n$ from the pool of n unique recovery delays, a new data set was created. Then noise was added to the peak intensities for each of the $3 \cdot n$ data points, according to a normal distribution with a standard deviation of $\varepsilon_{\mathrm{exp}}$. For every peak and saturation-recovery experiment, 1000 of such data sets containing $3 \cdot n$ randomly altered data points were created and fitted to the saturation recovery model as described by equation (5). The standard deviation ΔR_1 of all 1000 fitted R_1 parameters was then used as the error of R_1.
The sPRE is then obtained by performing a weighted linear regression using equation (7)

$$R_1(c) = \mathrm{sPRE} \cdot c + R_1^0 \qquad (7)$$

where c is the concentration of Omniscan, $R_1(c)$ is the fitted R_1 rate at the present of Omniscan with a concentration c, R_1^0 is the R_1 in the absence of Omniscan and sPRE is the slope and the desired sPRE

value. For the weighted linear regression, the previously determined errors ΔR_1 for R_1 was used, and the error of the concentration c was neglected.

Supplementary Figures

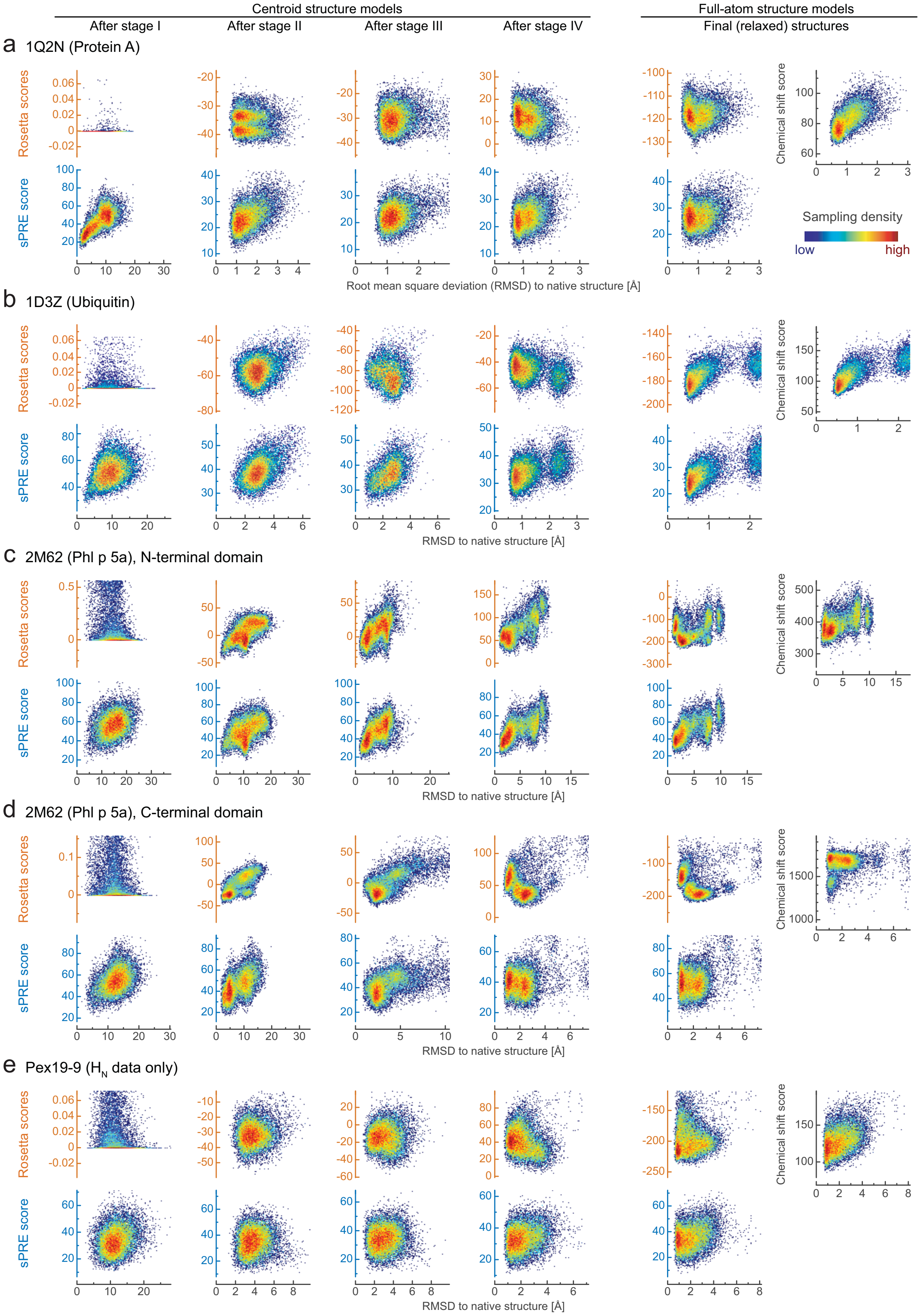

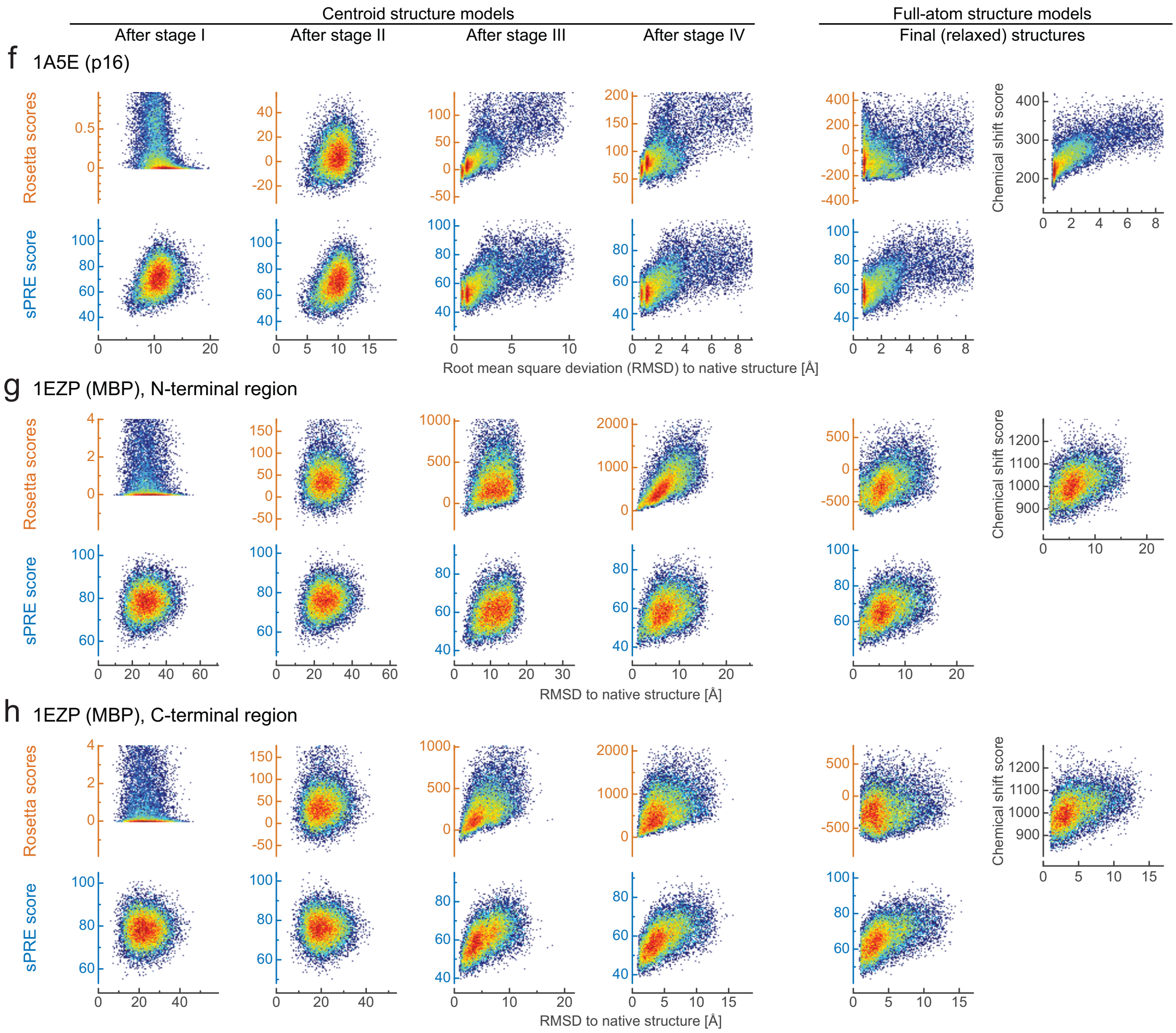

Supplementary figure 1. Scoring performance of the sPRE module. Ensembles of different proteins (a-i) have been generated and rescored using different scoring functions. Plots show 2D-histograms of the score and the C^{α}-RMSD to the native structure, with red corresponding to a high sampling density and dark blue corresponding to single structures. The ensembles contain centroid and full-atom models representing different stages of Rosetta's AbinitioRelax protocol (see column headers). Centroid models for Stages 1-4 were rescored using the corresponding Rosetta centroid score score0-3 (orange axis), the sPRE score (blue axis). Full-atom models were rescored using the Rosetta score score13_env_hb (orange axis), the chemical shift score (black axis) and the sPRE score (blue axis). Experimental sPRE data was used as listed in supplementary table 1.

a

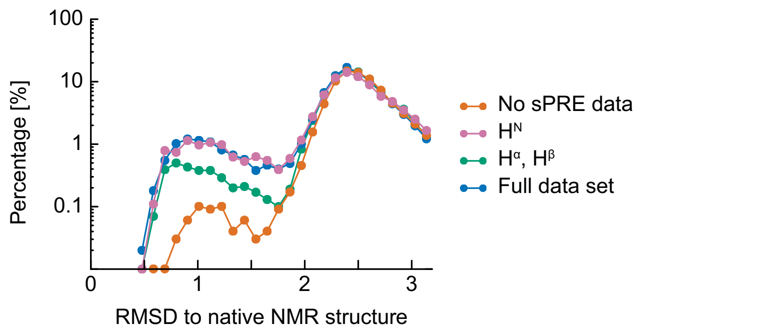

b

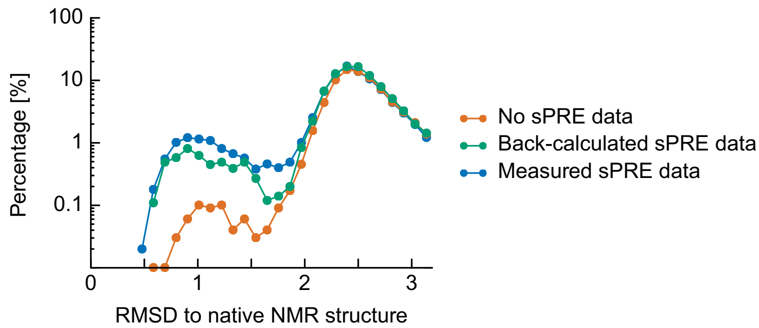

Supplementary figure 2. Redundancy of sPRE data for Ubiquitin. Plots show histograms of the C^{α}-RMSD distribution of different structure ensembles on a logarithmic scale. (a) Structure ensembles were generated using CS-Rosetta (orange) or sPRE-CS-Rosetta with different sPRE input data: the full experimental data set (blue), sPRE data of amide protons H^N only (magenta) and sPRE data of H^{α} and H^{β} protons only (green). (b) Structure ensembles were generated using CS-Rosetta (orange), sPRE-CS-Rosetta with 193 experimental sPRE values for H^N and $H^{aliphatic}$ protons (blue) and sPRE-CS-Rosetta with back-calculated sPRE data for all protons and carbon atoms (green).

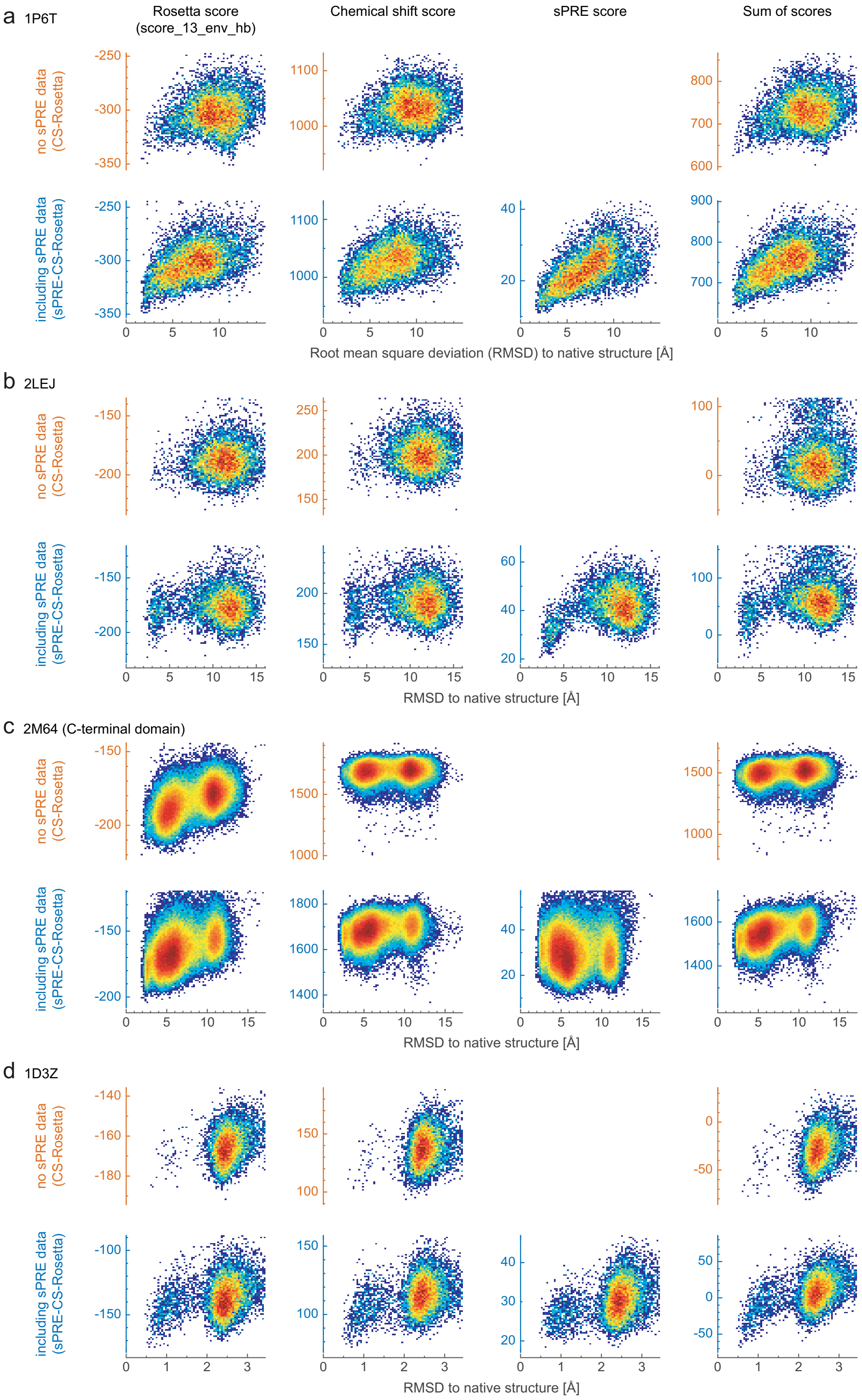

Supplementary figure 3. Performance of sPRE-CS-Rosetta. Comparison of structure ensembles obtained by CS-Rosetta (orange axis) and sPRE-CS-Rosetta (blue axis) for 1P6T (a), 2LEJ (b), 2M64 (c) and 1D3Z (d). Columns show different scores. From left to right: The full-atom Rosetta score (score13_env_hb), the chemical shift score, the sPRE score (for sPRE-CS-Rosetta runs only) and the sum of the first 2 (for CS-Rosetta) or the sum of all 3 scores (sPRE-CS-Rosetta). Plots show 2D-histograms of the respective score and the C$^{\alpha}$-RMSD to the native structure, with red corresponding to a high sampling density and dark blue corresponding to single structures.

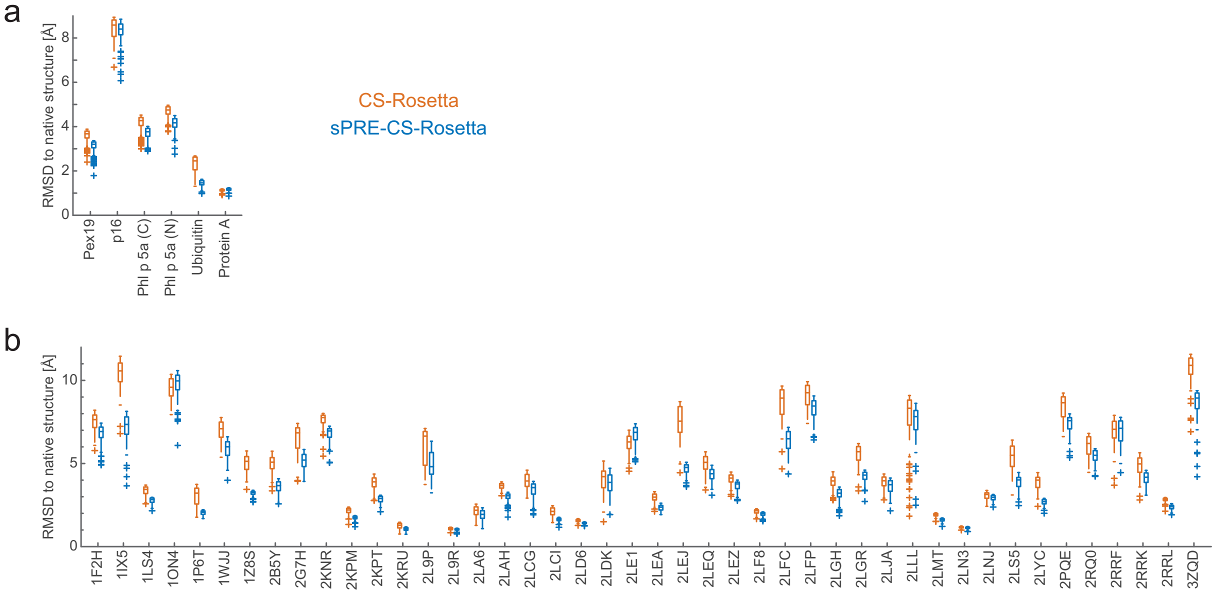

Supplementary figure 4. Effect of sPRE data on sampling efficiency. Structures for several proteins were predicted by CS-Rosetta (orange) and sPRE-CS-Rosetta (blue). For the sPRE module, experimental (a) and back-calculated (b) data was used. The 1% best structures by the C^{α}-RMSD to the native structure were filtered and the average C^{α}-RMSD of the subset was computed and plotted. Proteins for which the average C^{α}-RMSD was above 10 Å for CS-Rosetta and sPRE-CS-Rosetta are not shown (1CX1, 1GXE, 1RFL, 1XWE and 4A5V). All tested proteins are listed in supplementary table 2.

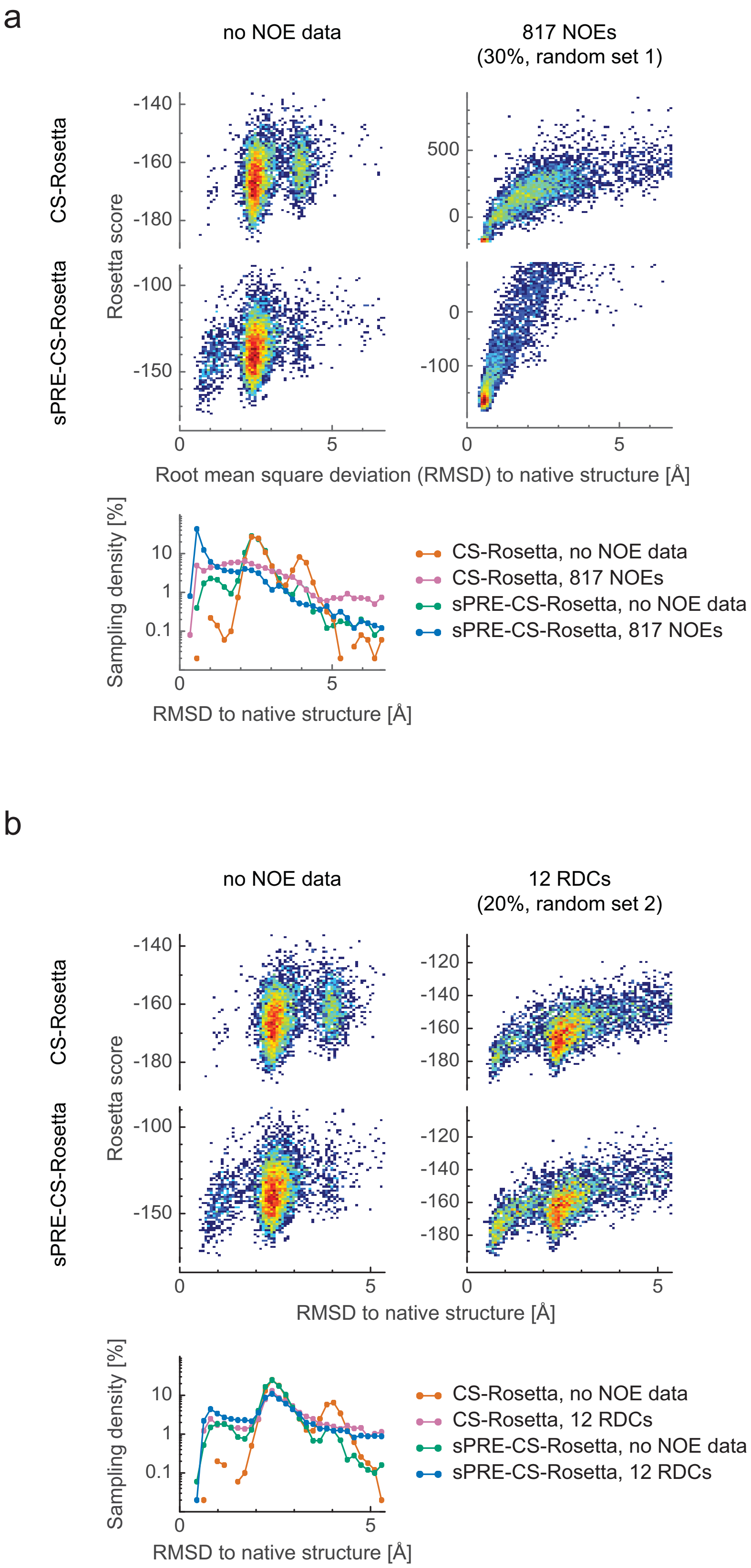

Supplementary figure 5. Sampling performance of sPRE-CS-Rosetta with NOE and RDC data. Structural models of ubiquitin (1D3Z) were predicted using classical CS-Rosetta as well as sPRE-CS-Rosetta in combination with experimental NOE (a) and RDC (b) data. Experimental sPRE data for H^N and $H^{aliphatic}$ protons was used for all computations and NOE and RDC restraints were obtained from the PDB entry 1D3Z. Plots show 2D-histograms of the respective score and the C^α-RMSD to the native structure, with red corresponding to a high sampling density and dark blue corresponding to single structures. Below the 4 plots, a logarithmic histogram shows the distribution the corresponding structures.

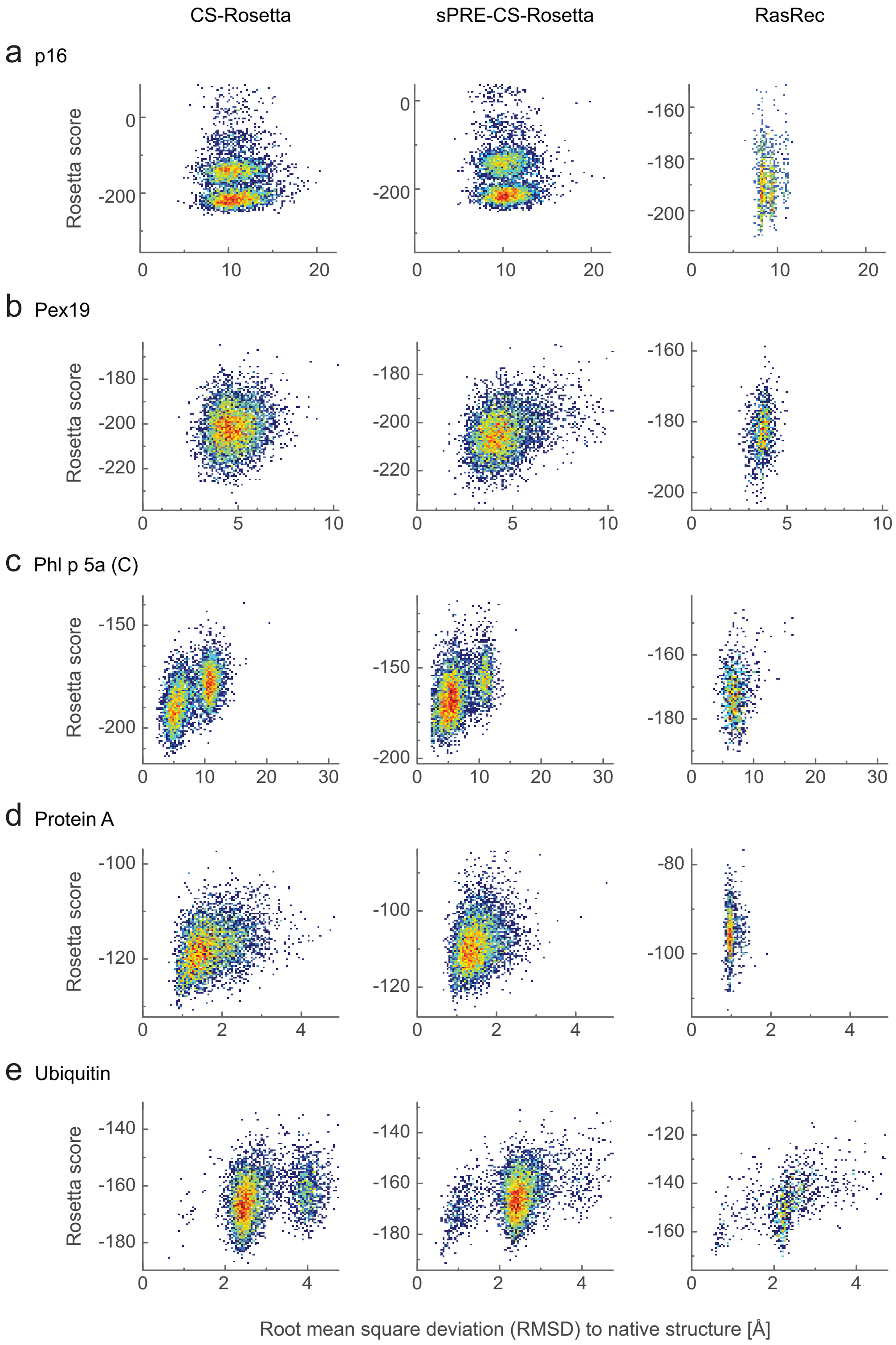

Supplementary figure 6. Comparison with RasRec-Rosetta. Structural ensembles of different proteins have been predicted using CS-Rosetta (left column), sPRE-CS-Rosetta (middle column) and RasRec-Rosetta (right column). Plots show 2D-histograms of the Rosetta score (score13_env_hb, in arbitrary units) and the C^{α}-RMSD to the native structure, with red corresponding to a high sampling density and dark blue corresponding to single structures. 5000 models are shown for CS-Rosetta and sPRE-CS-Rosetta, 1000 models for RasRec-Rosetta. Experimental data was used as input for sPRE-CS-Rosetta.

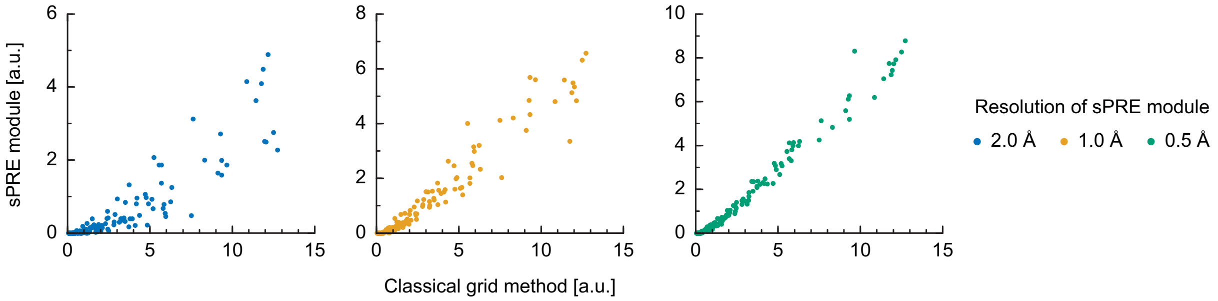

Supplementary figure 7. Verification of sPRE back-calculation and effect of the grid resolution on the accuracy. Back-calculated sPRE values for 1D3Z (Ubiquitin) using the sPRE module of Rosetta at different resolutions are compared to sPRE values computed using a classical grid-based approach with a resolution of 0.1A

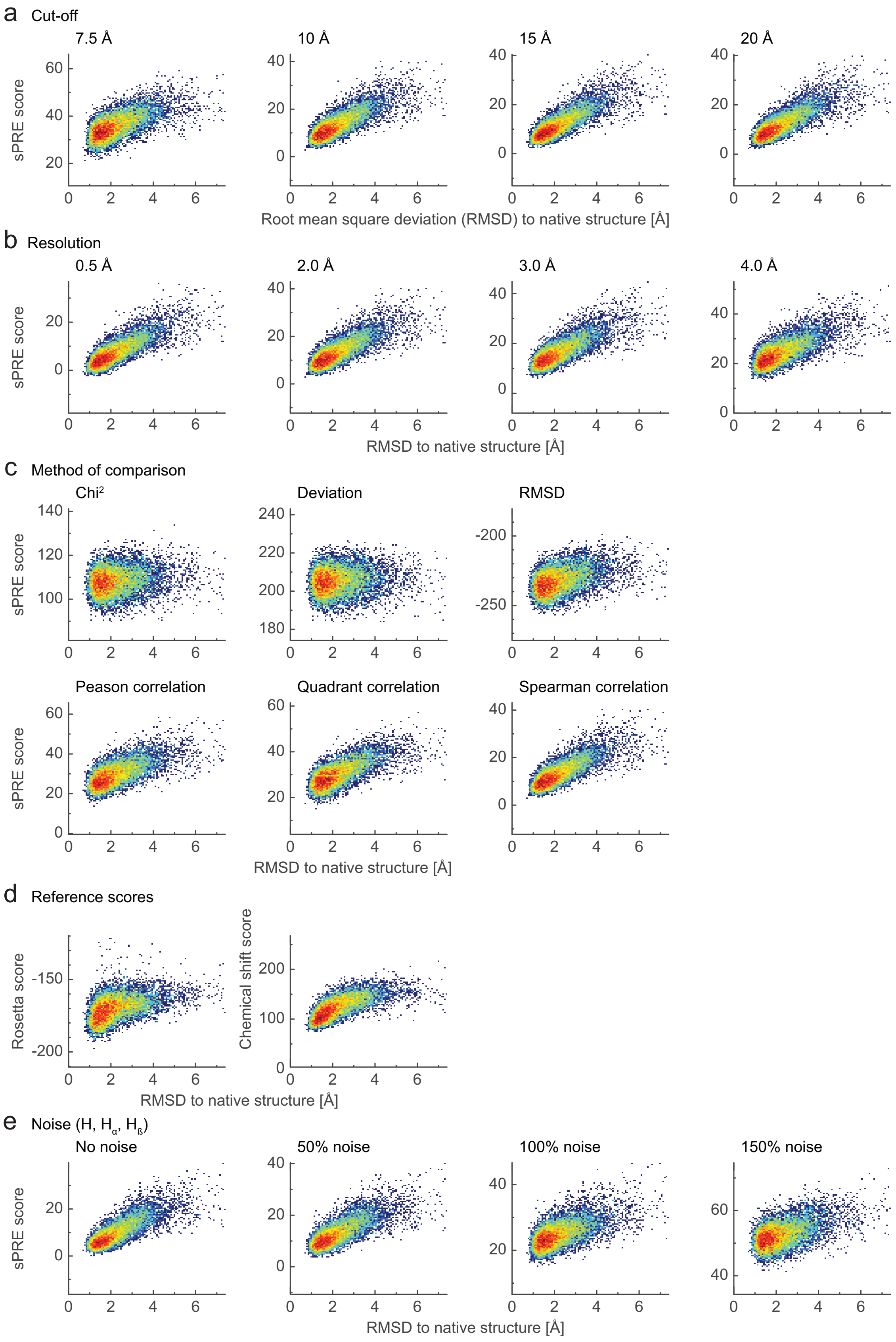

Supplementary figure 8. Effect of different scoring parameters. An ensemble for the DNA-Binding Domain of Ngtrf1 (2CKX) with 15000 full-atom structure models was obtained using classical CS-Rosetta and subsequently rescored using the sPRE score. Plots show 2D-histograms of the respective score and the C^{α}-RMSD to the native structure, with red corresponding to a high sampling density and dark blue corresponding to single structures. (a-c) The sPRE score was computed using back-calculated sPRE data and parameters of the sPRE score module were varied as indicated. (d, e) For comparison, the Rosetta full-atom score (score_13_env_hb) and the chemical shift score (d) as well as the effect of noise (e) are shown.

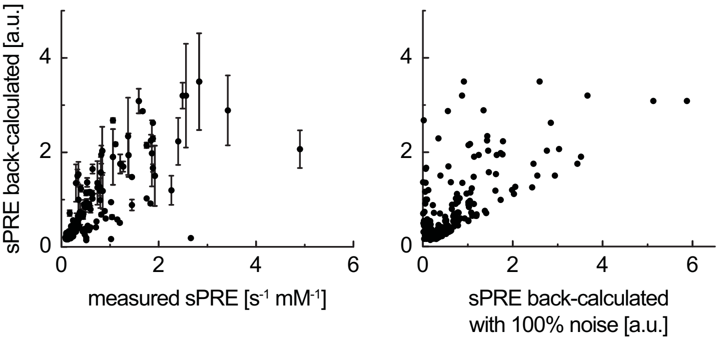

Supplementary figure 9. Agreement of experimental and back-calculated sPRE data. The correlation between measured sPRE data of ubiquitin (H^N and $H^{aliphatic}$ protons) and the back-calculated sPRE data is shown on the left. sPRE values were calculated using the published NMR ensemble (1D3Z) and error bars indicate the standard deviation between the 10 models of the NMR ensemble. In comparison, the effect of adding 100% noise to the back-calculated data is shown in the scatter plot on the right. Back-calculated sPRE values are shown in arbitrary units and scaled to fit the experimental values.

Supplementary Tables

Supplementary table 1. Proteins with experimental sPRE data used for benchmarking the sPRE module.

Protein	PDB	BMRB	Fold	Fragment used	sPRE data
Maltodextrin-Binding Protein (MBP)	1EZP	4986	α+β	1-370	H^N + $H^{aliphatic}$
Ubiquitin	1D3Z	6457	α+β	1-76	H^N + $H^{aliphatic}$
Z domain of protein A	1Q2N	4023	α	3-58	$H^α$ + $H^β$
Tumor suppressor p16INK4A	1A5E	4086	α	11-136	H^N + $H^{aliphatic}$
Major Grass Pollen Allergen Phl p 5a	2M64	19107	α	58-171 (N term. domain) 181-284 (C-term. domain)	H^N + $H^{aliphatic}$
Pex19	to be submitted	to be submitted	α	1-110	H^N

Supplementary table 2. Proteins with back-calculated sPRE data for benchmarking the sPRE module.

PDB	BMRB	Protein name	Number of residues	Fold
1CX1	4706	Second N-terminal cellulose-binding domain of β-1,4-Glucanase C	146	β
1F2H	4636	N-terminal domain of the TNFR1 associated protein	170	α+β
1GXE	5014	Central domain of cardiac myosin binding protein C	131	β
1IX5	4668	FK506-binding protein	152	α+β
1LS4	4814	Apolipophorin-III	157	α
1ON4	5742	Soluble domain of Sco1	170	α+β
1P6T	5813	Soluble region of P-type ATPase CopA	142	α+β
1RFL	5861	G-domain of MnmE protein	170	α
1WJJ	10090	Protein F20O9.120	127	α+β
1XWE	6001	C345C (NTR) domain of C5 of complement	148	α+β
1Z8S	6716	DnaB binding domain of DnaG (P16)	101	α
2B5Y	6603	Thioredoxin-like Protein	148	α+β
2G7H	7122	O6-Methylguanine DNA Methyltransferase	159	α+β
2KNR	16476	Ontario Center for Structural Proteomics target ATC0905	122	α+β
2KPM	16560	Northeast Structural Genomics Target NeR103A	83	α+β
2KPT	16569	Northeast Structural Genomics Consortium Target CgR26A	132	α+β
2KRU	16649	PCP_red domain of light-independent protochlorophyllide reductase subunit B	52	α
2L9P	17481	Northeast Structural Genomics Consortium Target SeR147	160	α+β
2L9R	17484	Homeobox domain of Homeobox protein Nkx-3.1	48	α
2LA6	17508	RRM domain of RNA-binding protein FUS	87	α+β
2LAH	17524	N-terminal domain of serine/threonine-protein kinase BUB1	151	α
2LCG	17611	Northeast Structural Genomics Consortium Target CrR115	137	α+β
2LCI	17613	Northeast Structural Genomics Consortium Target OR36	130	α+β
2LD6	17651	Histidine Phosphotransfer Domain of CheA	124	α
2LDK	17670	Northeast Structural Genomics Consortium Target AaR96	161	α+β
2LE1	17688	Northeast Structural Genomics Consortium Target TfR85A	144	α+β
2LEA	17705	RRM domain of Serine/arginine-rich splicing factor 2	88	α+β
2LEJ	17714	Prion protein mutant HuPrP	116	α
2LEQ	17723	Northeast Structural Genomics Consortium Target ChR145	146	α+β
2LEZ	17734	N-terminal domain of Secreted effector protein PipB2	116	α+β
2LF8	17742	ETS Domain of transcription factor ETV6	108	α+β
2LFC	17747	Subunit of fumarate reductase flavoprotein	146	α+β
2LFP	17768	Gp17 protein of bacteriophage SPP1	130	α+β
2LGH	17809	Northeast Structural Genomics Consortium Target AhR99.	139	α+β
2LJA	17927	C-terminal domain of putative thiol-disulfide oxidoreductase	137	α+β
2LLL	18053	C-terminal domain of Lamin-B2	111	β
2LMT	17353	N-terminal domain of Androcam	78	α+β
2LN3	18145	Northeast Structural Genomics Consortium Target OR135	76	α+β
2LNJ	18167	CyanoP subunit of photosystem II	159	α+β
2LS5	18411	Putative protein disulfide isomerase	151	α+β
2LYC	18717	C-terminal domain of Spindle and kinetochore-associated protein 1	129	α+β
2PQE	15232	Proline-free mutant of staphylococcal nuclease	138	α+β
2RQ0	11062	Lipocalin-type Prostaglandin D Synthase	158	α+β
2RRK	11422	E. coli ORF135 protein	136	α+β
2RRL	11423	C-terminal domain of the FliK	97	α+β
2LGR	15593	Human protein C6orf130	141	α+β
2RRF	11251	C-terminal region of Zinc finger FYVE domain-containing protein 21	125	α+β
3ZQD	17701	B. subtilis L,D-transpeptidase	168	α+β
4A5V	18039	N-terminal apple domains of Toxoplasma gondii microneme protein 4	160	α+β

Supplementary table 3a. Effect of assignment completeness and noise level on Rosetta sampling. Structural models of 2LEJ were predicted using simulated sPRE data of different quality. The percentages of models with a C^{α}-RMSD of 5 Å or less to the native structure are shown for different assignment completeness, noise levels and different protein nuclei.

No sPRE data

	1.2%

Using simulated H^N sPRE data

	100% assignment	70% assignment	40% assignment
Noise +/-30%	6.4%	5.0%	2.3%
Noise +/-60%	6.9%	4.6%	2.2%
Noise +/-100%	4.4%	4.2%	1.6%
Noise +/-200%	1.7%	0.4%	0.9%
Noise +/-400%	0.0%	0.0%	0.0%

Using simulated H^N, $H^{methyl\text{-}ILV}$ sPRE data

	100% assignment	70% assignment	40% assignment
Noise +/-30%	4.0%	3.9%	7.0%
Noise +/-60%	4.4%	4.0%	6.2%
Noise +/-100%	1.7%	3.4%	3.9%
Noise +/-200%	2.3%	0.7%	0.1%
Noise +/-400%	0.8%	0.0%	0.3%

Using simulated H^N, H^{α}, H^{β} sPRE data

	100% assignment	70% assignment	40% assignment
Noise +/-30%	7.4%	4.4%	2.2%
Noise +/-60%	9.3%	4.7%	1.6%
Noise +/-100%	11.2%	4.8%	1.3%
Noise +/-200%	3.1%	4.7%	0.4%
Noise +/-400%	1.9%	0.9%	0.2%

Supplementary table 3b. Effect of assignment completeness and noise level on Rosetta sampling. Structural models of 1P6T were predicted using simulated sPRE data of different quality. The percentages of models with a C^{α}-RMSD of 5 Å or less to the native structure are shown for different assignment completeness, noise levels and different protein nuclei.

No sPRE data

	3.5%

Using simulated H^N sPRE data

	100% assignment	70% assignment	40% assignment
Noise +/-30%	25.8%	23.8%	20.7%
Noise +/-60%	26.1%	22.1%	20.7%
Noise +/-100%	27.2%	25.1%	13.9%
Noise +/-200%	15.9%	10.8%	0.1%
Noise +/-400%	12.1%	2.3%	0.6%

Using simulated H^N, $H^{methyl\text{-}ILV}$ sPRE data

	100% assignment	70% assignment	40% assignment
Noise +/-30%	28.9%	30.6%	22.3%
Noise +/-60%	29.8%	27.2%	23.2%
Noise +/-100%	30.1%	32.9%	10.4%
Noise +/-200%	16.2%	7.4%	0.9%
Noise +/-400%	2.3%	2.9%	0.4%

Using simulated H^N, H^{α}, H^{β} sPRE data

	100% assignment	70% assignment	40% assignment
Noise +/-30%	28.1%	27.5%	18.9%
Noise +/-60%	26.4%	26.9%	17.1%
Noise +/-100%	22.9%	28.7%	14.7%
Noise +/-200%	19.7%	16.7%	12.9%
Noise +/-400%	3.3%	10.9%	3.6%

Supplementary table 3c. Effect of assignment completeness and noise level on Rosetta sampling. Structural models of 1LS4 were predicted using simulated sPRE data of different quality. The percentages of models with a C^{α}-RMSD of 5 Å or less to the native structure are shown for different assignment completeness, noise levels and different protein nuclei.

No sPRE data

	5.9%

Using simulated H^N sPRE data

	100% assignment	70% assignment	40% assignment
Noise +/-30%	27.3%	21.8%	10.9%
Noise +/-60%	28.4%	14.7%	7.0%
Noise +/-100%	26.3%	7.6%	2.3%
Noise +/-200%	2.0%	2.1%	0.1%
Noise +/-400%	0.3%	0.0%	0.0%

Using simulated H^N, $H^{methyl\text{-}ILV}$ sPRE data

	100% assignment	70% assignment	40% assignment
Noise +/-30%	31.3%	40.9%	10.6%
Noise +/-60%	30.8%	41.0%	10.3%
Noise +/-100%	28.0%	41.0%	9.8%
Noise +/-200%	13.7%	43.2%	0.1%
Noise +/-400%	4.4%	2.8%	0.0%

Using simulated H^N, H^{α}, H^{β} sPRE data

	100% assignment	70% assignment	40% assignment
Noise +/-30%	44.6%	48.9%	43.7%
Noise +/-60%	46.1%	49.9%	45.4%
Noise +/-100%	46.0%	52.6%	33.1%
Noise +/-200%	29.8%	39.0%	6.8%
Noise +/-400%	2.0%	10.6%	2.4%

Supplementary table 3d. Effect of assignment completeness and noise level on Rosetta sampling. Structural models of 1Z8S were predicted using simulated sPRE data of different quality. The percentages of models with a C^{α}-RMSD of 5 Å or less to the native structure are shown for different assignment completeness, noise levels and different protein nuclei.

No sPRE data

	0.6%

Using simulated H^N sPRE data

	100% assignment	70% assignment	40% assignment
Noise +/-30%	14.1%	12.3%	12.0%
Noise +/-60%	13.2%	9.5%	6.4%
Noise +/-100%	3.8%	3.7%	1.2%
Noise +/-200%	0.2%	0.1%	0.0%
Noise +/-400%	0.1%	0.3%	0.0%

Using simulated H^N, $H^{methyl\text{-}ILV}$ sPRE data

	100% assignment	70% assignment	40% assignment
Noise +/-30%	32.1%	28.9%	6.0%
Noise +/-60%	30.8%	26.5%	5.8%
Noise +/-100%	21.2%	16.2%	2.6%
Noise +/-200%	4.8%	0.7%	0.0%
Noise +/-400%	0.2%	0.0%	0.0%

Using simulated H^N, H^{α}, H^{β} sPRE data

	100% assignment	70% assignment	40% assignment
Noise +/-30%	38.6%	28.6%	15.0%
Noise +/-60%	38.2%	26.6%	13.0%
Noise +/-100%	35.7%	25.9%	18.2%
Noise +/-200%	24.4%	7.4%	4.4%
Noise +/-400%	2.6%	3.6%	0.0%

Supplementary table 4a. Sampling performance of sPRE-CS-Rosetta with NOE data. Structural models of ubiquitin (1D3Z) were predicted using classical CS-Rosetta as well as sPRE-CS-Rosetta in combination with different sets of experimental NOE data. Experimental sPRE data for H^{N} and $H^{aliphatic}$ protons was used for all computations and NOE restraints were obtained from the PDB entry 1D3Z. The percentages of models with a C^{α}-RMSD of 1 Å or less to the native structure are shown for the full set of NOEs, in the absence of NOE data and with randomly drawn subsets of NOEs varying in size (between 27 and 1363 NOEs). To account for selection effects, 3 to 4 different random sets were generated for every NOE pool size.

		no NOEs	27 NOEs (1%)	54 NOEs (2%)	136 NOEs (5%)	272 NOEs (10%)	817 NOEs (30%)	1363 NOEs (50%)	2726 NOEs (full set)
Set 1	no sPRE	0.2%	0.9%	10.8%	33.4%	60.0%	10.5%	5.0%	31.1%
	sPRE	3.1%	2.3%	24.7%	62.6%	79.9%	58.8%	31.9%	45.3%
Set 2	no sPRE		0.3%	7.4%	12.4%	31.9%	56.7%	61.0%	
	sPRE		1.8%	17.5%	72.6%	77.8%	83.6%	82.4%	
Set 3	no sPRE		1.9%	2.4%	20.2%	35.0%	48.1%	20.0%	
	sPRE		6.7%	7.3%	29.3%	73.3%	79.7%	82.6%	
Set 4	no sPRE		4.3%	23.1%					
	sPRE		9.8%	31.2%					

Supplementary table 4b. Sampling performance of sPRE-CS-Rosetta with NOE data. The same ensembles as in supplementary table 4a were used and the percentages of models with a C^{α}-RMSD of 4 Å or more to the native structure are shown.

		no NOEs	27 NOEs (1%)	54 NOEs (2%)	136 NOEs (5%)	272 NOEs (10%)	817 NOEs (30%)	1363 NOEs (50%)	2726 NOEs (full set)
Set 1	no sPRE	10.9%	7.6%	8.8%	10.7%	4.0%	31.1%	19.8%	23.4%
	sPRE	4.9%	13.9%	0.5%	2.2%	1.2%	5.2%	5.9%	3.9%
Set 2	no sPRE		4.5%	2.0%	12.3%	6.8%	10.6%	8.4%	
	sPRE		5.1%	1.5%	1.9%	1.8%	1.2%	2.9%	
Set 3	no sPRE		14.6%	5.1%	6.5%	22.3%	7.5%	12.4%	
	sPRE		1.5%	3.7%	3.4%	4.8%	1.8%	2.7%	
Set 4	no sPRE		0.4%	0.3%					
	sPRE		1.9%	0.4%					

Supplementary table 5. Sampling performance of sPRE-CS-Rosetta with RDC data. Structural models of ubiquitin (1D3Z) were predicted using classical CS-Rosetta as well as sPRE-CS-Rosetta in combination with different sets of experimental RDC data. Experimental sPRE data for H^N and $H^{aliphatic}$ protons was used for all computations and H^N-N RDC restraints in one medium were obtained from the PDB entry 1D3Z. The percentages of models with a C^α-RMSD of 1 Å or less to the native structure are shown for the full set of RDCs, in the absence of RDC data and with randomly drawn subsets of RDCs varying in size (between 6 and 47 H^N-N RDCs). To account for selection effects, 2 to 4 different random sets were generated for every RDC pool size.

		no RDC	6 RDCs	12 RDCs	18 RDCs	25 RDCs	31 RDCs	47 RDCs	63 RDCs (full set)
Set 1	no sPRE	0.17%	0.58%	1.70%	0.46%	1.60%	3.52%	2.66%	4.48%
	sPRE	3.12%	2.76%	3.04%	1.02%	2.42%	4.16%	3.32%	4.92%
Set 2	no sPRE		0.10%	4.78%	0.76%	0.88%	2.26%	2.92%	
	sPRE		1.58%	8.70%	1.32%	1.72%	3.06%	3.05%	
Set 3	no sPRE		0.26%	2.06%					
	sPRE		1.70%	4.40%					
Set 4	no sPRE		0.48%	5.42%					
	sPRE		2.04%	10.18%					

Supplementary table 6. Proteins used to optimize the parameters for the sPRE module.

Protein	PDB	Fold	Fragment	sPRE data
UBA domain of p62	1Q02	α	1-52	synthetic
Protein RPA3401	2JTV	α+β	1-64	synthetic
Protein Atu4866	2JMB	β	1-79	synthetic
DNA-Binding Domain of Ngtrf1	2CKX	α	578-660	synthetic
RRM-1 of Yeast NPL3	2OSQ	α+β	9-79	synthetic
Protein MJ1198	2K52	β	1-74	synthetic

Supplementary table 7. Tested candidates for computing the sPRE score according to $s_{\mathrm{score}_j} = A_j \cdot \tilde{s}_{\mathrm{score}_j} - B_j$. Different methods to compare the experimental to the back-calculated sPRE data were implemented according to equation (3). A_j and B_j are the scaling and offset constants for the scoring function score_j, n is the number of atoms for which a reference sPRE value is supplied, i is the index of the atom, $\mathrm{sPRE}_i^{\mathrm{exp}}$ and $\mathrm{sPRE}_i^{\mathrm{model}}$ are the reference and back-calculated sPRE value of the i-th atom, r_i^{exp} and r_i^{model} are the ranks of the reference and back-calculated sPRE value of the i-th atom, $|\cdot|$ denotes the absolute value, $\overline{\cdot}$ is the average of the corresponding quantity over all n atoms, and sgn(·) is the sign function.

s_{score_j}	$\tilde{s}_{\mathrm{score}_j}$	A_j	B_j
s_{spearman}	$1 - \frac{\sum_{i=1}^{n}\left(\left(r_i^{\mathrm{model}} - \overline{r^{\mathrm{model}}}\right) \cdot \left(r_i^{\mathrm{exp}} - \overline{r^{\mathrm{exp}}}\right)\right)}{\sqrt{\sum_{i=1}^{n}\left(r_i^{\mathrm{model}} - \overline{r^{\mathrm{model}}}\right)^2 \cdot \sum_{i=1}^{n}\left(r_i^{\mathrm{exp}} - \overline{r^{\mathrm{exp}}}\right)^2}}$	1.906	0.144
s_{pearson}	$1 - \frac{\sum_{i=1}^{n}\left(\left(\mathrm{sPRE}_i^{\mathrm{model}} - \overline{\mathrm{sPRE}^{\mathrm{model}}}\right) \cdot \left(\mathrm{sPRE}_i^{\mathrm{exp}} - \overline{\mathrm{sPRE}^{\mathrm{exp}}}\right)\right)}{\sqrt{\sum_{i=1}^{n}\left(\mathrm{sPRE}_i^{\mathrm{model}} - \overline{\mathrm{sPRE}^{\mathrm{model}}}\right)^2 \cdot \sum_{i=1}^{n}\left(\mathrm{sPRE}_i^{\mathrm{exp}} - \overline{\mathrm{sPRE}^{\mathrm{exp}}}\right)^2}}$	1.906	0.144
s_{quadrant}	$1 - \frac{1}{n}\sum_{i=1}^{n}\left(\mathrm{sgn}\left(\mathrm{sPRE}_i^{\mathrm{model}} - \overline{\mathrm{sPRE}^{\mathrm{model}}}\right) \cdot \mathrm{sgn}\left(\mathrm{sPRE}_i^{\mathrm{exp}} - \overline{\mathrm{sPRE}^{\mathrm{exp}}}\right)\right)$	1.906	0.144
s_{chi}	$\sqrt{\frac{1}{n} \cdot \sum_{i=1}^{n}\frac{\left(\mathrm{sPRE}_i^{\mathrm{model}} - \mathrm{sPRE}_i^{\mathrm{exp}}\right)^2}{\mathrm{sPRE}_i^{\mathrm{exp}}}}$	95	130
s_{chi2}	$\frac{1}{n} \cdot \sum_{i=1}^{n}\frac{\left(\mathrm{sPRE}_i^{\mathrm{model}} - \mathrm{sPRE}_i^{\mathrm{exp}}\right)^2}{\mathrm{sPRE}_i^{\mathrm{exp}}}$	35	67
$s_{\mathrm{meanDeviation}}$	$\frac{1}{n} \cdot \sum_{i=1}^{n}\left\|\mathrm{sPRE}_i^{\mathrm{model}} - \mathrm{sPRE}_i^{\mathrm{exp}}\right\|$	95	130
$s_{\mathrm{meanDeviation4}}$	$\frac{1}{n} \cdot \sum_{i=1}^{n}\sqrt[4]{\mathrm{sPRE}_i^{\mathrm{model}} - \mathrm{sPRE}_i^{\mathrm{exp}}}$	80	78
s_{rmsd}	$\sqrt{\frac{1}{n}\sum_{i=1}^{n}\left(\mathrm{sPRE}_i^{\mathrm{model}} - \mathrm{sPRE}_i^{\mathrm{exp}}\right)^2}$	118	390
s_{msd}	$\frac{1}{n} \cdot \sum_{i=1}^{n}\left(\mathrm{sPRE}_i^{\mathrm{model}} - \mathrm{sPRE}_i^{\mathrm{exp}}\right)^2$	10	93

Supplementary table 8. Critical parameters of the sPRE module and recommended values.

Parameter	Description	Recommended value
Grid resolution	Dimension of each cell of the grid	1 - 2 Å
Integration	Cutoff radius, above which all sPRE contributions are neglected	10 Å
Scaling	Global weight of the sPRE score	67<br>(scaling of sPRE score in Rosetta framework assumed to be 1.0)
Weights	Stage-specific weight of the sPRE score in the AbinitioRelax protocol	1-1-1-1-1<br>(equal weights in all stages)
Method of comparison	Algorithm comparing the predicted and the experimental sPRE data	Spearman correlation coefficient

Supplementary table 9. Effect of different weights of the sPRE score on Rosetta sampling. Structure models of different proteins were predicted using different weights of the sPRE score and the corresponding percentages of models with a maximum C^{α}-RMSD of 4 Å (for 1Q02, 2JTV and 2JMB) or 1.5 Å (for 2CKX, 2OSQ and 2K52) to the native structure are shown.

Scaling (a.u.)	1Q02	2JMB	2JTV	2CKX	2K52	2OSQ
0 (Reference)	10%	40%	70%	22%	0.26%	5.9%
3	14%	44%	78%	22%	0.27%	6.2%
10	29%	54%	87%	22%	0.45%	6.2%
33	74%	67%	97%	25%	0.55%	7.3%
67	93%	74%	98%	31%	0.99%	8.5%
100	96%	74%	95%	34%	0.99%	9.5%
150	94%	69%	72%	32%	1.08%	11.0%
300	78%	42%	20%	17%	0.59%	11.1%

Supplementary table 10. Effect of stage-specific weights of the sPRE score on Rosetta sampling. Structure models of different proteins were predicted using varying weights of the sPRE score for the different Abinitio stages and the corresponding percentages of models with an maximum C^{α}-RMSD of 4 Å (for 1Q02, 2JTV and 2JMB) or 1.5 Å (for 2CKX, 2OSQ and 2K52) to the native structure are shown.

Weight in stage										
I	II	III	IIIa	IV	1Q02	2JMB	2JTV	2CKX	2K52	2OSQ
0	0	0	0	0	10%	40%	70%	22%	0.26%	5.9%
1	0	0	0	0	10%	41%	70%	24%	0.29%	7.1%
0	1	0	0	0	11%	42%	72%	31%	0.23%	5.3%
0	0	1	0	0	11%	44%	73%	23%	0.30%	7.3%
0	0	0	1	0	10%	48%	73%	19%	0.35%	7.5%
0	0	0	0	1	92%	60%	98%	28%	1.38%	11.9%
0.3	1	1	1	0.3	53%	63%	94%	26%	0.41%	5.4%
1	1	1	1	1	93%	74%	98%	31%	0.99%	8.5%

Supplementary table 11. Effect of different correlation coefficients on Rosetta sampling. Structure models of different proteins were predicted using different correlation coefficients to compare the back-calculated to the given input sPRE data. The corresponding percentages of models with a maximum C^{α}-RMSD of 4 Å (for 1Q02, 2JTV and 2JMB) or 1.5 Å (for 2CKX, 2OSQ and 2K52) to the native structure are shown.

Correlation coefficient	1Q02	2JMB	2JTV	2CKX	2K52	2OSQ
No sPRE data (Reference)	10%	40%	70%	22%	0.26%	5.9%
Correlation	84%	48%	97%	26%	0.60%	10.7%
Quadrant correlation	63%	52%	94%	28%	0.68%	6.4%
Spearman correlation	93%	74%	98%	31%	0.99%	8.5%

Supplementary table 12. Runtime for scoring a centroid ensemble. 3000 centroid structures of Ubiquitin were scored using a combination of the sPRE score and a Rosetta centroid score (see columns). Settings for the sPRE back-calculation were changed as indicated (res. resolution, int. radius of integration). The user space runtime was recorded and compared to only scoring with the Rosetta centroid score (100%, top row).

Settings for sPRE back-calculation	Rosetta score0	Rosetta score1	Rosetta score2	Rosetta score3
No sPRE score	100%	100%	100%	100%
sPRE score, res. 2 Å, int. 10 Å	168%	166%	166%	166%
sPRE score, res. 1 Å, int. 10 Å	182%	179%	178%	178%
sPRE score, res. 0.5 Å, int. 10 Å	262%	254%	253%	253%
sPRE score, res. 0.2 Å, int. 10 Å	1507%	1439%	1433%	1430%
sPRE score, res. 2 Å, int. 15 Å	173%	170%	170%	169%
sPRE score, res. 2 Å, int. 20 Å	180%	177%	176%	176%
sPRE score, res. 2 Å, int. 30 Å	197%	193%	192%	192%
sPRE score, res. 2 Å, int. 40 Å	227%	220%	219%	219%

Supplementary table 13. Runtime comparison for structure determination. Classical CS-Rosetta AbinitioRelax runs were started for Ubiquitin and Protein A. For the same proteins, CS-Rosetta runs with activated sPRE score were started and the runtime per obtained structure was compared to the reference CS-Rosetta run. Settings for the sPRE back-calculation were changed as indicated (res. resolution, int. radius of integration).

Settings for sPRE back-calculation	Ubiquitin	Protein A
Reference CS-Rosetta	100%	100%
sPRE-CS-Rosetta, res. 2 Å, int. 10 Å	191%	217%
sPRE-CS-Rosetta, res. 1 Å, int. 10 Å	546%	683%
sPRE-CS-Rosetta, res. 0.5 Å, int. 10 Å	2670%	3417%
sPRE-CS-Rosetta, res. 2 Å, int. 20 Å	400%	482%
sPRE-CS-Rosetta, res. 2 Å, int. 30 Å	910%	1086%

References

[1] a) H. G. Hocking, K. Zangger, T. Madl, *Chemphyschem* **2013**, *14*, 3082-3094; b) T. Madl, W. Bermel, K. Zangger, *Angew. Chem. Int. Ed.* **2009**, *48*, 8259-8262; c) G. Pintacuda, G. Otting, *J. Am. Chem. Soc.* **2002**, *124*, 372-373.

[2] H. M. Berman, J. Westbrook, Z. Feng, G. Gilliland, T. N. Bhat, H. Weissig, I. N. Shindyalov, P. E. Bourne, *Nucleic Acids Res.* **2000**, *28*, 235-242.

[3] O. F. Lange, D. Baker, *Proteins* **2012**, *80*, 884-895.

[4] E. L. Ulrich, H. Akutsu, J. F. Doreleijers, Y. Harano, Y. E. Ioannidis, J. Lin, M. Livny, S. Mading, D. Maziuk, Z. Miller, E. Nakatani, C. F. Schulte, D. E. Tolmie, R. Kent Wenger, H. Yao, J. L. Markley, *Nucleic Acids Res.* **2008**, *36*, D402-408.

[5] F. Delaglio, S. Grzesiek, G. W. Vuister, G. Zhu, J. Pfeifer, A. Bax, *J. Biomol. NMR* **1995**, *6*, 277-293.

[6] B. A. Johnson, R. A. Blevins, *J. Biomol. NMR* **1994**, *4*, 603-614.

[7] W. F. Vranken, W. Boucher, T. J. Stevens, R. H. Fogh, A. Pajon, M. Llinas, E. L. Ulrich, J. L. Markley, J. Ionides, E. D. Laue, *Proteins* **2005**, *59*, 687-696.

[8] M. Tashiro, R. Tejero, D. E. Zimmerman, B. Celda, B. Nilsson, G. T. Montelione, *J. Mol. Biol.* **1997**, *272*, 573-590.

[9] A. Tevelev, I. J. Byeon, T. Selby, K. Ericson, H. J. Kim, V. Kraynov, M. D. Tsai, *Biochemistry* **1996**, *35*, 9475-9487.

[10] J. J. Helmus, C. P. Jaroniec, *J. Biomol. NMR* **2013**, *55*, 355-367.
